# LncRNA NKX2‐1‐AS1 promotes tumor progression and angiogenesis via upregulation of SERPINE1 expression and activation of the VEGFR‐2 signaling pathway in gastric cancer

**DOI:** 10.1002/1878-0261.12911

**Published:** 2021-02-13

**Authors:** Fei Teng, Ju‐Xiang Zhang, Yi Chen, Xiao‐Dong Shen, Chang Su, Yan‐Jiao Guo, Pu‐Hua Wang, Chen‐cheng Shi, Ming Lei, Yi‐Ou Cao, Shao‐Qun Liu

**Affiliations:** ^1^ Department of Gastrointestinal Surgery Minhang Hospital Fudan University Shanghai China; ^2^ Institute of Fudan‐Minhang Academic Health System Minhang Hospital Fudan University Shanghai China; ^3^ Shanghai Med‐X Engineering Center for Medical Equipment and Technology School of Biomedical Engineering Shanghai Jiao Tong University China

**Keywords:** gastric cancer, competing endogenous RNA, serpin family E member 1, VEGFR‐2 signaling pathway, NKX2‐1 antisense RNA 1

## Abstract

Long noncoding RNAs (lncRNAs) can compete with endogenous RNAs to modulate the gene expression and contribute to oncogenesis and tumor metastasis. lncRNA NKX2‐1‐AS1 (NKX2‐1 antisense RNA 1) plays a pivotal role in cancer progression and metastasis; however, the contribution of aberrant expression of NKX2‐1‐AS1 and the mechanism by which it functions as a competing endogenous RNA (ceRNA) in gastric cancer (GC) remains elusive. NKX2‐1‐AS1 expression was detected in paired tumor and nontumor tissues of 178 GC patients by quantitative reverse transcription PCR (qRT‐PCR). Using loss‐of‐function and gain‐of‐function experiments, the biological functions of NKX2‐1‐AS1 were evaluated both *in vitro* and *in vivo*. Further, to assess that NKX2‐1‐AS1 regulates angiogenic processes, tube formation and co‐culture assays were performed. RNA binding protein immunoprecipitation (RIP) assay, a dual‐luciferase reporter assay, quantitative PCR, Western blot, and fluorescence in situ hybridization (FISH) assays were performed to determine the potential molecular mechanism underlying this ceRNA. The results indicated that NKX2‐1‐AS1 expression was upregulated in GC cell lines and tumor tissues. Overexpression of NKX2‐1‐AS1 was significantly associated with tumor progression and enhanced angiogenesis. Functionally, NKX2‐1‐AS1 overexpression promoted GC cell proliferation, metastasis, invasion, and angiogenesis, while NKX2‐1‐AS1 knockdown restored these effects, both *in vitro* and *in vivo*. RIP and dual‐luciferase assays revealed that the microRNA miR‐145‐5p is a direct target of NKX2‐1‐AS1 and that NKX2‐1‐AS1 serves as a ceRNA to sponge miRNA and regulate angiogenesis in GC. Moreover, serpin family E member 1 (SERPINE1) is an explicit target for miR‐145‐5p; besides, the NKX2‐1‐AS1/miR‐145‐5p axis induces the translation of SERPINE1, thus activating the VEGFR‐2 signaling pathway to promote tumor progression and angiogenesis. NKX2‐1‐AS1 overexpression is associated with enhanced tumor cell proliferation, angiogenesis, and poor prognosis in GC. Collectively, NKX2‐1‐AS1 functions as a ceRNA to miR‐145‐5p and promotes tumor progression and angiogenesis by activating the VEGFR‐2 signaling pathway via SERPINE1.

## Graphical abstract

This study revealed that NKX2‐1‐AS1 might serve as an independent prognostic biomarker in gastric cancer (GC). NKX2‐1‐AS1 promotes cell proliferation and tumor angiogenesis in GC. Moreover, NKX2‐1‐AS1 directly targets miR‐145‐5p to upregulate SERPINE1 and promote tumor progression and angiogenesis in a VEGFR‐2‐dependent manner.

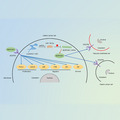

**Abbreviations**
AJCC: American Joint Committee on Cancer
ATAD2: AAA domain containing 2
ceRNA: competing endogenous RNA
DIG: digoxigenin
FC: fold change
FDR: false discovery rate
FISH: fluorescence in situ hybridization
GC: gastric cancer
HEK: human embryonic kidney
HRP: horseradish peroxidase
H‐score: histoscore
HUVECs: human umbilical vein endothelial cells
IHC: immunohistochemistry
KIF23: kinesin family member 23
lncRNAs: long noncoding RNAs
MACC1‐AS1: MACC1 antisense RNA 1
MEST: mesoderm‐specific transcript
miRNAs: microRNAs
NC: negative control
NKX2‐1‐AS1: NKX2‐1 antisense RNA 1
PAI‐1: plasminogen activator inhibitor‐1
PFS: progression‐free survival
PI: propidium iodide
SERPINE1: serpin family E member 1
shRNAs: short‐hairpin RNAs
STAD: stomach adenocarcinoma
TCGA: The Cancer Genome Atlas
TMEM100: transmembrane protein 100
TRIB3: tribbles pseudokinase 3
uPA: urokinase‐type plasminogen activator
uPAR: cellular receptor for urokinase‐type plasminogen activator

## 1 Introduction

Gastric cancer (GC) ranks the fifth most frequent malignancy with over 1.22 million diagnosed GC cases annually and represents the third leading cause of cancer‐associated mortality (nearly 865 000 deaths) worldwide, constituting and reported in 2017 [1]. East Asia, particularly China and Japan [2], contributes to more than half of the global burden of stomach cancer. Due to the lack of early symptoms, most GC patients are diagnosed in the advanced stages at the time of diagnosis [3]. Despite substantial advances in GC diagnosis and management of advanced‐stage GC over the recent decades, the five‐year survival rate for GC patients with the advanced disease continues to be poor [4, 5]. Notably, GC tumor‐associated angiogenesis is the predominant event associated with an increased risk of tumor recurrence and unfavorable prognosis [6]. Thus, an enhanced understanding of the molecular mechanisms underlying tumor angiogenesis represents the key to prevent recurrence or progression and facilitate early detection and prediction of therapeutic outcomes in patients with GC.

Long noncoding RNAs (lncRNAs) represent a class of RNA polymerase II transcripts of > 200 nucleotides that are not translated into proteins [7]. Increasing evidence in recent years suggested that lncRNAs are intimately involved in regulating multiple processes underlying cancer development [8, 9]. Moreover, the dysregulated expression of lncRNAs has been implicated in multiple cancer types and shown to be differentially expressed across various tumor differentiation stages. Unlike microRNAs (miRNAs), lncRNAs exhibit extremely complicated mechanisms to regulate gene expression; notably, lncRNA can interact with DNA, RNA, and proteins to modulate transcriptional machinery assembly [10]. Accumulating studies have also indicated that some lncRNAs can act as miRNA sponges and competitively inhibit the biological functions of miRNAs [11, 12]. The lncRNA NKX2‐1‐AS1 was identified to be highly expressed in primary lung adenocarcinomas and found to be associated with lung carcinoma cell migration [13]. Furthermore, bioinformatics studies have revealed that NKX2‐1‐AS1 may act as a competing endogenous RNA (ceRNA) in GC [14]. However, the contribution of aberrant expression of NKX2‐1‐AS1 in GC tumorigenesis and its prognostic relevance in GC remains elusive.

The plasminogen activation system, comprising the urokinase‐type plasminogen activator (uPA), the cellular receptor for uPA (uPAR), and its specific inhibitor plasminogen activator inhibitor‐1 (PAI‐1 or SERPINE1), is recognized to play a vital role in tumor progression and angiogenesis [15, 16]. Numerous studies demonstrated that the association of SERPINE1 overexpression with tumor progression and unfavorable outcomes in various cancers, including GC [17, 18]. Aberrant expression of SERPINE1 has been observed in numerous cancer types and is associated with poor prognosis. In particular, the role of SERPINE1 in tumor angiogenesis has been intensively investigated. Studies with SERPINE1‐deficient mice indicated that host SERPINE1 expression is crucial for tumor angiogenesis [19, 20, 21], and its effects are mediated by the modulation of endothelial cell plasmin‐mediated proteolysis [22], migration [23, 24], and apoptosis [25]. Emerging studies have demonstrated that the inhibition of SERPINE1 with SK‐216 (a specific PAL‐1 inhibitor) can reduce the extent of angiogenesis in the tumors and suppress progression *in vivo* angiogenesis. Moreover, *in vitro* studies have demonstrated suppression of VEGF‐induced migration and tube formation by human umbilical vein endothelial cells (HUVECs) [26]. Based on these findings, it was speculated that the aberrant expression of SERPINE1 might be associated with GC progression due to increased tumor angiogenesis promoted by SERPINE1.

## 2 Materials and methods

### 2.1 Data mining and analysis

RNAseq dataset of GC patients was retrieved from The Cancer Genome Atlas (TCGA) database (https://portal.gdc.cancer.gov). The following inclusion criteria were applied to the present study, (1) histopathologically confirmed diagnosis of stomach adenocarcinoma (STAD); (2) access to sufficient data for analysis; (3) no diagnosis of other malignancies or distant metastases before surgery; (4) no previous adjuvant radiation or chemotherapy before surgery. A total of 373 samples, comprising of 343 STAD tissues and 30 adjacent noncancerous gastric tissues, were included in this analysis. This study was conducted in accordance with the publication guidelines provided by the TCGA database (www.cancergenome.nih.gov/publications/publicationlines).

### 2.2 Identification of differentially expressed RNAs

The level three mRNA expression profiles of the STAD patients and corresponding clinical data were obtained from the TCGA‐STAD dataset (up to Sep. 1, 2017). TCGA database provided the normalized RNA sequencing data, including lncRNA, miRNA, and mRNA profiles from STAD patients. Data from each sample included the RNAseq, miRNAseq, and corresponding clinical data. We compared the STAD patients and adjacent noncancerous tissues to identify the differential expression of lncRNA, miRNA, and mRNA by using the edgeR package of R software [27] (version 3.4.3, 2017, https://www.r‐project.org) with a rigorous threshold as log2 fold change (FC) of > 2 and a false discovery rate (FDR)‐adjusted P‐value of < 0.01 were considered to be differentially expressed (DElncRNA, DEmiRNA, and DEmRNA, respectively). Heatmaps and volcano plots of DElncRNAs, DEmRNAs, and DEmiRNAs were generated using the pheatmap (www.rdocumentation.org/packages/pheatmap) and ggplot2 (www.ggplot.yhathq.com/) packages in R software [28] and performed the hierarchical clustering analysis to distinguish statistically significant differentially expressed RNAs (DERNAs). Kyoto Encyclopedia of Genes and Genomes (KEGG) pathway enrichment analyses were performed to detect the function of the DEmRNAs with the clusterProfiler and visualized using ggplot2 packages in R. The association between the DEmRNAs, DElncRNAs, and DEmiRNAs in the ceRNA network and the overall survival (OS) of STAD patients was analyzed by survival and qvalue packages in R. Statistical significance was set at *P* < 0.05.

### 2.3 Construction of the ceRNA network

To determine putative lncRNA‐miRNA‐mRNA interactions, we considered only the pool of dysregulated lncRNAs and mRNAs. Then, we identified the miRNA seed sequences that function as shared (overlapping) binding sites for these lncRNAs and mRNAs. lncRNA‐miRNA interaction was predicted using the miRcode (www.mircode.org/), a comprehensive, searchable map of putative microRNA target sites in the long noncoding transcriptome. The target genes of DEmiRNAs, and the miRNA‐mRNA binding site were predicted using the starBase v2.0 (http://starbase.sysu.edu.cn/). Subsequently, the target DEmRNAs of DEmiRNA were predicted using miRDB (www.mirdb.org), TargetScan (www.targetscan.org), and miRTarBase (mirtarbase.mbc.nctu.edu.tw) databases. To further enhance the ceRNA network's reliability, we selected lncRNAs, which potentially interacted with mRNAs in the miRNA predictions and TCGA‐STAD differential expression data. Then, cytoscape (v3.5.0; www.cytoscape.org) was used to visualize and construct the ceRNA network [29].

### 2.4 Gastric cancer patients and tissue specimens

For the present study, a total of 178 samples, consisting of GC tissues and their matched adjacent nontumor tissues, were obtained from GC patients who underwent surgical resections at Minhang Hospital, Fudan University, between 2011 and 2019. None of the patients received radiation therapy or chemotherapy prior to surgery. Only histopathologically confirmed cases were included in this study. However, patients who received chemo‐ or radiotherapy before surgery and those with incomplete clinical data were excluded from the study. For all patients, pathological stages were determined according to the seventh edition American Joint Committee on Cancer (AJCC) manual cancer staging system using available clinical and pathologic tumor, node, and metastasis data. The histopathological examination was performed for all the specimens by two experienced pathologists. The fresh tissue specimens were immediately frozen in liquid nitrogen and stored at −80 °C until subsequent RNA extraction. The present study was approved by the Human Research Ethics Review Board of Minhang Hospital, and informed consent was also obtained from all patients.

### 2.5 Gastric cancer cell lines and cell culture

The human GC cell lines (BGC‐823, AGS, MGC‐803, HGC‐27, MKN‐45, and SGC‐7901), the immortalized gastric normal mucosal cell line (GES‐1), human umbilical vein endothelial cells (HUVECs), and human embryonic kidney (HEK) 293 FT cells were procured from the Chinese Academy of Sciences (CAS) (Shanghai) Cell Bank. All cell lines were cultured in RPMI‐1640 medium (Hyclone, Logan, UT, USA) supplemented with 10% fetal bovine serum (FBS; Invitrogen) in a humidified atmosphere of 5% CO_2_ at 37 °C. When the cells reached a confluence of > 90%, cells were passaged and reseeded at an appropriate density.

### 2.6 Quantitative real‐time PCR

Total RNA was extracted from GC tissues and matched adjacent nontumor tissues and cells using TRIzol Reagent (Invitrogen, Carlsbad, CA, USA) following the manufacturer's protocol. The concentration and purity of total RNA were measured on a Nanodrop spectrophotometer (Thermo Scientific, Waltham, MA, USA). The high‐quality cDNA was synthesized using the Moloney Leukemia Virus Reverse Transcriptase Kit (Promega, Madison, WI, USA) following the manufacturer's protocols. qRT‐PCR was performed to measure lncRNA and mRNA expression with SYBR Green Mix (Promega) following the manufacturer's instructions. The GAPDH was used as an internal reference. miRNA expression was measured by qRT‐PCR using the All‐in‐One™ miRNA qRT‐PCR kit (GeneCopoeia, Carlsbad, CA, USA) and ABI 7500 fast real‐time PCR system (Applied Biosystems, Waltham, MA, USA). U6 snRNA was used as an internal reference gene. The relative gene expression as fold change was calculated using the 2^−△△CT^. All experiments were performed in triplicate. Primer sequences used in this study are listed in Table S1.

### 2.7 Immunohistochemistry

SERPINE1 protein expression was determined by immunohistochemistry (IHC), and the extent of immunological staining was semi‐quantitatively analyzed as described previously. The formalin‐fixed, paraffin‐embedded (FFPE) tissue samples were sectioned at a thickness of 5 μm thickness. In brief, tissue sections were deparaffinized in xylene and rehydrated in an ethanol gradient. Antigen retrieval was performed by heating the tissue sections at 100 °C in sodium citrate buffer for 20 min. Subsequently, the sections were incubated with anti‐CD34 (cat.3569S, CST 1:300) primary antibody at 4°C overnight, then incubated with anti‐HRP secondary antibody (Dako, Glostrup, Denmark) at room temperature for 2 h. The sections were stained with Mayer's hematoxylin solution and examined under a microscope. Two independent pathologists performed IHC staining scoring. The staining intensity was scored as follows: 0 for negative staining, 1 for weakly positive, 2 for moderately positive, and 3 for strongly positive. The positive proportion of stained tumor cells was scored as follows: ≤5% positive cells (score of 0+), 6% to 25% positive cells (score of 1+), 26% to 50% positive cells (score of 2+), ≥51% positive cells (score of 3+). To evaluate the mean staining intensity of tumor samples, at least 100 cells from multiple tumor areas were analyzed. Cytoplasmic expression was scored for staining intensity and proportion of tissue stained using a Histoscore (H‐score, from 0 to 300), using the formula: Histoscore = ∑(*I* × Pi), where *I* = Intensity, Pi = percentage of positively stained tumor cells [30]. Scoring was performed by two independent pathologists, blinded to the clinical outcomes. For animal studies, subcutaneous tumors were harvested from nude mice, fixed in formalin, embedded in paraffin, sectioned, and stained as described in the IHC section.

### 2.8 Cell proliferation and colony formation assays

Cell proliferation was determined using the Cell counting kit‐8 (CCK‐8; Dojindo, Tokyo, Japan) assay. Briefly, tumor cells were seeded into 96‐well plates at 24 h post‐transfection and incubated for 7 days. Subsequently, cells were incubated for an additional 4 h with 10 μL of CCK‐8. Cell proliferation was measured by absorbance at 450 nm with a microplate reader. For colony formation, tumor cells were seeded into 6‐well plates at a density of 500 cells/well at 24 h post‐transfection and incubated at 37 °C with 5% CO_2_. After 2 weeks, cells were fixed with methanol and stained with 0.1% crystal violet for 1 h at RT. Colonies were counted using Quantity One (Bio‐Rad, Hercules, CA, USA). All experiments were performed triplicate, and the mean values were calculated.

### 2.9 Lentivirus production and cell transfection

Two lentivirus vectors containing NKX2‐1‐AS1 short‐hairpin RNAs (shRNAs) were purchased from GenePharma (Shanghai, China) and transfected into GC cell lines. NKX2‐1‐AS1‐overexpressing or SERPINE1‐overexpressing pLVX‐IRES‐ZsGreen vector was purchased from Takara Bio (Catalog no. 632187, CA, USA). shRNA and empty vector were transfected into the GC cell lines, respectively. At 48 h post‐transfection, cells were incubated in 2 μg/mL puromycin for 2 weeks for selecting transfected cells with stable knockdown or overexpression of NKX2‐1‐AS1. Transfection efficiency was quantified by qRT‐PCR. The knockdown efficiencies of shNKX2‐1‐AS1‐1 in AGS and HGC‐27 were 81% and 76%, respectively; the knockdown efficiencies of shNKX2‐1‐AS1‐2 in AGS and HGC‐27 were 55% and 75%, respectively. Hsa‐miR‐145‐5p mimic, hsa‐miR‐145‐5p inhibitor, and negative control (NC) oligonucleotides were procured from Ribobio (Guangzhou, China). GC cell lines were transiently transfected with the above‐mentioned oligonucleotides and plasmids using Lipofectamine 3000 (Invitrogen), following the manufacturer's instructions.

### 2.10 Cell migration and invasion assays

To detect the migration and invasion potential, a transwell migration assay (8‐μm pore size; Corning Costar, Cambridge, MA, USA) was performed. Briefly, nonadherent tumor cells cultured in serum‐free RPMI‐1640 medium were seeded into the upper chamber, and RPMI‐1640 medium supplemented with 20% FBS was added into the lower chamber as a chemoattractant. After 24 h to 48 h of incubation, the inserted membranes were then fixed in methanol and stained with 0.1% crystal violet (Beyotime Institute of Biotechnology, Shanghai, China). The upper surface of the membranes was gently wiped, and cells migrating to the bottom surface of the membrane were counted and observed under the microscope. Similarly, the cell invasion assay was performed, but with the addition of a layer of Matrigel on the upper side of the transwell membrane.

### 2.11 Cell cycle and cell apoptosis

For cell cycle analysis, cells were seeded in 6‐well plates at a density of 3 × 10^5^ cells/well and incubated overnight at 37 °C. Adherent cells were collected and washed twice with PBS. After fixed in cold 70% ethanol at 4 °C overnight, the cells were incubated with 10 mg/mL RNase and 1 mg/mL propidium iodide (PI) according to the manufacturer's instructions. The cell cycle was analyzed by flow cytometry (BD Biosciences, San Diego, CA, USA), and the percentage of cells in the G0/G1, S, and G2/M phases was determined using Cell Quest acquisition software (BD Biosciences). For apoptosis analysis, the annexin V/propidium iodide assay was performed according to the manufacturer's instructions. In brief, cells were cultured in 2 mL culture medium in 6‐well plates at 37 °C for 18–24 h. Cells were then collected, washed twice in cold PBS, centrifuged, and resuspended in 100 µL binding buffer (BD Pharmingen) containing 5 µL Annexin V‐FITC and 5 µL PI solution for 20 min in the dark. The stained cells were analyzed by flow cytometry (FACS Calibur, BD Biosciences, San Jose, CA, USA) within 30 min after staining. The following criteria distinguished different subpopulations: Q1 (Annexin V‐negative but PI‐positive) as necrotic cells; Q2 (Annexin V/PI‐double‐positive) as of late apoptotic cells; Q3 (Annexin V/PI‐double negative), as live cells; Q4 (Annexin V‐positive but PI‐negative), as early apoptotic cells. The apoptotic rate was determined as the percentage of early apoptotic (Q4) + late apoptotic (Q2).

### 2.12 *In vivo* tumor formation

All the animal experiments were approved by the Animal Ethics Committee of Fudan University. Mice were cared for in accordance with the National Institute of Health (NIH) Guide for the Care and Use of Laboratory Animals. Four‐week‐old female BALB/c nude mice were purchased from Shanghai SLAC Laboratory Animal Co., Ltd. Mice were housed under specific pathogen‐free conditions with a 12‐h light/dark cycle and provided *ad libitum* access to tap water and food. AGS cells transfected with the NKX2‐1‐AS1 vector or the empty vector or AGS cells transfected with the shNKX2‐1‐AS1‐1 or shRNA‐NC were subcutaneously injected into the right axillary of BALB/c nude mice. Tumor volume was measured once every 4 days using a Vernier caliper. Mice were monitored daily for tumor growth and sacrificed 4 weeks postinjection. Tumors and major organs were harvested, weighed, and analyzed by qRT‐PCR and IHC.

### 2.13 Fluorescence *in situ* hybridization

The AGS cells were fixed in 4% paraformaldehyde (PFA) at room temperature for 15 min, followed by PBS wash. Cells were then permeabilized with 0.5% Triton X‐100 at 4 °C for 15 min. Subsequently, cells were incubated with digoxigenin (DIG)‐labeled NKX2‐1‐AS1 probes or Control‐FISH probes at 55°C for 4 h and washed with 2X PBS for 10 min. The signal was detected by horseradish peroxidase (HRP)‐conjugated anti‐DIG secondary antibodies (Jackson, West Grove, PA, USA). DAPI was used to counterstain nuclear. Olympus confocal laser scanning microscope was used to obtain the images.

### 2.14 Luciferase reporter assays

The reporter vector pmirGLO‐NKX2‐1‐AS1‐WT was constructed by cloning NKX2‐1‐AS1 cDNA, which contained a predictive binding site of miR‐145‐5p, into the pmirGLO Dual‐Luciferase miRNA Target Expression Vector (Promega). The mutant NKX2‐1‐AS1 containing point mutations of the miR‐145‐5p seed region binding site was specifically synthesized and inserted into the above‐mentioned vector, which was designated as pmirGLO‐NKX2‐1‐AS1‐Mut. HEK‐293FT cells were cultured into 96‐well plates and co‐transfected with pmirGLO‐NKX2‐1‐AS1–3’‐UTR vectors, including wild‐type or mutant fragments and miR‐145‐5p, mimic, and the pmirGLO vector was used as the NC. To confirm the direct interaction of miR‐145‐5p and SERPINE1, wild‐type and mutant SERPINE1 3’‐UTR fragments were amplified by qRT‐PCR and cloned into the pmirGLO vector (Promega) using the one‐step directed cloning kit (Novoprotein, Shanghai, China); the resultant vectors were designated as SERPINE1‐WT and SERPINE1‐Mut, respectively. The miR‐145‐5p mimic was co‐transfected with SERPINE1‐WT or SERPINE1‐Mut vector into HEK‐293FT cells using Lipofectamine 3000 (Invitrogen). Using the Dual‐Luciferase Reporter Assay System (Promega), the luciferase assay was performed at 48 h post‐transfection according to the manufacturer's instructions. The relative luciferase activity was normalized to Renilla luciferase activity as a control for transfection efficiency. The primers used for vector construction were provided in Additional file 1: Table S1. Data were presented as the mean ± standard deviation (SD) of three independent experiments performed in triplicate.

### 2.15 RNA binding protein immunoprecipitation (RIP) analysis

The RIP assay was performed using a Magna RIP RNA binding protein immunoprecipitation kit (Merck Millipore, Billerica, MA, USA) according to the manufacturer's instructions. AGS and HGC‐27 cells were lysed in RIP buffer and then coupled with Ago2 antibody (ab32381; Abcam Inc., Cambridge, MA, USA) or IgG antibody (ab172730; 1 : 5000; Abcam Inc.) and incubated with magnetic beads. Subsequently, the immunoprecipitated RNA was subjected to RT‐qPCR.

### 2.16 *In vitro* tube formation assay

Using Matrigel's tube formation assay, the angiogenic potential of HUVEC cells was evaluated based on their capacity to form tube‐like structures *in vitro* [31]. Briefly, HUVEC cells were seeded in DMEM with 10% FBS for 24 h prior to transfection. Then, NKX2‐1‐AS1‐overexpressing vector and shNKX2‐1‐AS1–1 were transfected into HUVEC for 24 h, as described previously. Then, cells were harvested and cultured on the Matrigel‐coated plates for 24 h. Calcein‐AM (cat. C326, Dojindo, Japan) was used to stain cells. Tube formation was evaluated by fluorescence microscopy. ImageJ (version 1.52a) was used to quantify the tube length and number of branches.

### 2.17 Western blot assay

Total protein was extracted from the cultured cells (1 × 10^6^) on ice using RIPA buffer (Cell Signaling Technology, Danvers, MA, USA) supplemented with a protease inhibitors cocktail. Protein concentrations were measured with the BCA Protein Assay Kit (KeyGEN BioTECH, Nanjing, China). Total cytoplasmic and nucleic proteins were extracted using the PROTTOT‐1KT ProteoPrep kit (Sigma‐Aldrich, St. Louis, MO, USA). Equal amounts (30 µg) of protein from individual samples were separated using SDS/PAGE and transferred the proteins onto a polyvinylidene difluoride (PVDF) membrane. Membranes were blocked with 5% BSA in PBS. Subsequently, the membrane was incubated with primary antibodies followed by incubation with the corresponding secondary antibodies. The target bands were visualized using the Chemiluminescence Western Lightning Plus‐ECL kit (PerkinElmer, Waltham, Massachusetts, USA). Following primary antibodies were used: anti‐SERPINE1 (cat.11907, CST; 1 : 1000), anti‐VEGFR‐2 (cat.9698, CST; 1 : 1000), anti‐p‐VEGFR‐2 (cat.2478, CST; 1 : 1000), anti‐PLC‐λ (cat.5690, CST; 1 : 1000), anti‐p‐PLC‐λ (cat.8713, CST; 1 : 1000), anti‐Erk1/2 (cat.4695, CST; 1 : 1000), anti‐p‐Erk1/2 (cat.4370, CST; 1 : 1000), anti‐P38 (cat.8690, CST; 1 : 1000), anti‐p‐P38 (cat.4511, CST; 1 : 1000), anti‐FAK (cat.71433, CST; 1 : 1000), anti‐p‐FAK (cat.8556, CST; 1 : 1000), anti‐Src (cat.2109, CST; 1 : 1000), anti‐p‐Src (cat.6943, CST; 1 : 1000), anti‐Akt (cat.4685, CST; 1 : 1000), anti‐p‐Akt (cat.4060, CST; 1 : 1000), anti‐GAPDH (cat.51332, CST; 1 : 1000), and HRP‐conjugated goat anti‐rabbit (cat.7074, CST; 1 : 3000) or anti‐mouse IgG (cat.7076, CST; 1 : 3000) antibodies.

### 2.18 Statistical analysis

All statistical analyses were performed using spss 23.0 (SPSS, Chicago, IL, USA) and graphpad prism 7 (GraphPad Prism, Inc., La Jolla, CA, USA). Data are expressed as the mean ± SD of three independent experiments unless otherwise indicated. A two‐tailed Student *t*‐test was used to analyze the differences between the two groups. Comparisons among three or more groups were assessed using one‐way analysis of variance (ANOVA) followed by post hoc test through Student–Newman–Keuls test. A chi‐square test or Fisher's exact test was used to evaluate the differences between NKX2‐1‐AS1 expression and clinicopathological characteristics. Survival analysis was performed using the Kaplan–Meier method and was compared with the log‐rank test. All tests were two‐tailed, and a *P*‐value of < 0.05 was considered statistically significant.

## 3 Results

### 3.1 Differentially Expressed RNAs (DERNAs) in GC

**Fig. 1:**
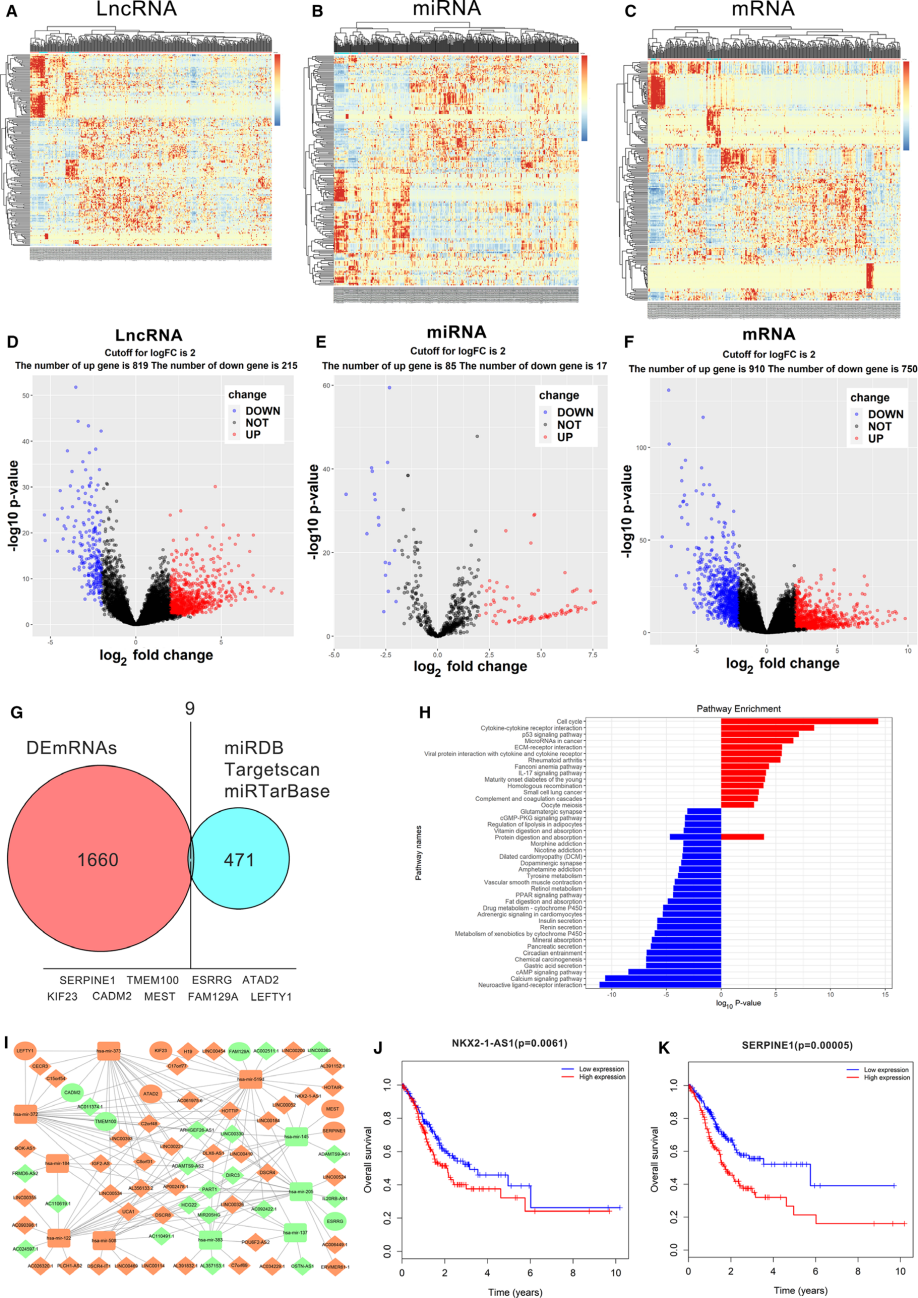
RNAseq data analysis of STAD samples retrieved from the TCGA database. (A–C) Clustered heat maps of differentially expressed RNAs (DERNAs) in STAD tissues and adjacent nontumor tissues. Individual rows represent distinct RNAs, and columns represent distinct samples. Differentially expressed lncRNAs, miRNAs, and mRNAs, in STAD and adjacent non‑tumor tissues are defined by the standard of log2FC > 2 and FDR < 0.01. FC, folds change; FDR, false discovery rate. (D–F) Volcano plots are used to evaluate changes in the expression levels of (D) long noncoding RNAs, (E) microRNAs, and (F) mRNAs between GC tissues and normal adjacent tissues. The values of the *x*‐ and *y*‐axes indicate the normalized signal values of the group (log scaled). (G) Identification of 471 commonly differentially expressed mRNAs is defined as the targeted of 29 DEmiRNAs from three publicly profile datasets (miRDB, Targetscan, and miRTarBase). The shared areas represent the most commonly altered mRNAs between DEmRNAs and target mRNAs, which include SERPINE1. (H) Enrichment analysis of differentially expressed mRNAs, based on the KEGG, Kyoto Encyclopedia of Genes and Genomes (KEGG) pathway databases (bar plot shows the enrichment scores of significantly enriched KEGG pathways). (I) lncRNA–miRNA–mRNA ceRNA network. Green squares indicate downregulated miRNAs; green circles indicate downregulated mRNAs; and green diamonds indicate downregulated lncRNAs. Orange squares indicate upregulated miRNAs; orange circles indicate upregulated mRNAs; and orange diamonds indicate upregulated lncRNAs. (J, K) Kaplan‑Meier survival curves for NKX2‐1‐AS1 and SERPINE1.

The RNA‐Seq dataset of 343 STAD patients and 30 adjacent nontumor samples were included in the present study from the TCGA‐STAD database; 1660 DEmRNAs (910/54.8% upregulated and 750/45.2% downregulated), 102 miRNAs (85/83.3% upregulated and 17/16.7% downregulated) and 1034 lncRNAs (819/79.2% upregulated and 215/20.8% downregulated) were identified (Fig. 1A–F). Using miRDB, Targetscan, and miRTarBase, we determined the potential binding miRNAs of all DEmRNAs, and considered the intersection of these potential binding miRNAs and DEmiRNAs, and identified 29 DEmiRNAs as the research objective. Then, the 471 mRNAs that these 29 DEmiRNAs can bind to and a total of 1660 DEmRNAs were selected to intersect, and finally, 9 DEmRNAs were obtained as the research objective. Nine intersecting mRNAs interacting with 10 DEmiRNAs were selected of the 471 predicted target mRNAs and 1660 DEmRNAs and used to construct the ceRNA network (Fig. 1G). To further elucidate the potential functional implication of these 1660 DEmRNAs, KEGG pathway enrichment analysis was performed. In addition, 15 pathways associated with upregulated DEmRNAs and 28 pathways related to downregulated DEmRNAs were identified. Notably, the up‐ and downregulated DEmRNAs were primarily enriched in biological processes (BP) and associated with cell cycle and neuroactive ligand‐receptor interaction, respectively (Fig. 1H). To better understand the mechanisms by which lncRNAs bind to miRNAs and regulate mRNA expression in GC, a lncRNA–miRNA–mRNA regulatory axis was extracted from the ceRNA network involving 62 DElncRNAs, 10 DEmiRNAS, and 9 DEmRNAs constructed by Cytoscape 3.5.0 (Fig. 1I). Of note, some DEmRNAs including Tribbles Pseudokinase 3 (TRIB3), Serpin Family E Member 1 (SERPINE1), Mesoderm Specific Transcript (MEST), Kinesin Family Member 23 (KIF23), Transmembrane Protein 100 (TMEM100), and AAA Domain Containing 2 (ATAD2) in the ceRNA network were previously reported to be associated with cancer. Next, the association between the differentially expressed genes in the ceRNA network and the survival outcomes of GC patients was analyzed to identify the potential prognostic factors. We found that 9 of the 62 DElncRNAs and 2 of the 9 DEmRNAs, including NKX2‐1‐AS1 and SERPINE1, were significantly associated with OS (Fig. 1J,K, and data not shown). Given that the NKX2‐1‐AS1/miR145‐5p/SERPINE1 was the only ceRNA regulatory axis that revealed a negative association with OS at both its lncRNA and mRNA levels and that miR‐145‐5p was expressed at a low level in cancer tissues, our data suggested that the NKX2‐1‐AS1/miR‐145‐5p/SERPINE1 axis might have a potential role in predicting the prognosis of GC patients.

### 3.2 Overexpression of NKX2‐1‐AS1 in GC

**Fig. 2:**
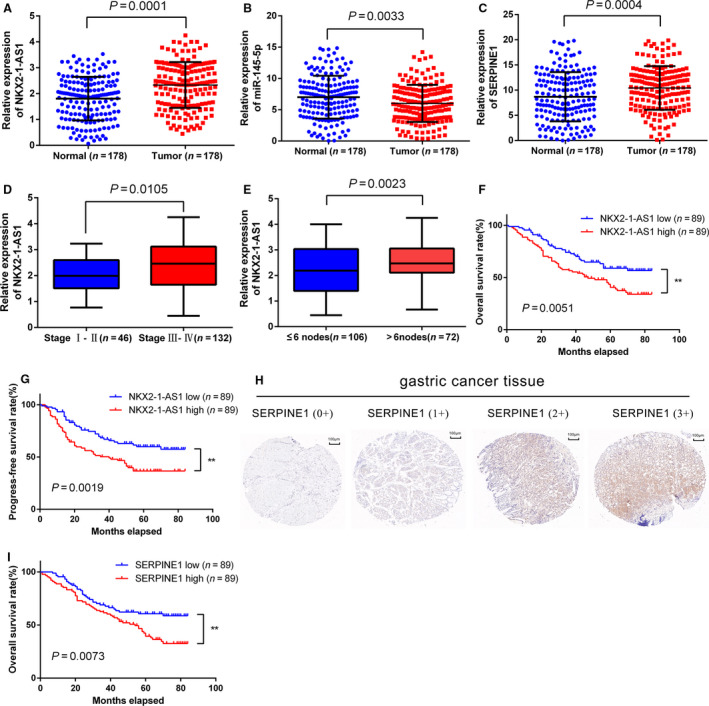
Association of NKX2‐1‐AS1 and SERPINE1 overexpression with the progression and prognosis of GC. (A–C) Expression of NKX2‐1‐AS1, miR‐145‐5p, and SERPINE1 detected by qRT‐PCR in 178 paired GC tissues and adjacent nontumor tissues. Data are presented as the relative expression in tumor tissues relative to adjacent nontumor tissues. (D, E) Relative expression of NKX2‐1‐AS1 in GC with different TNM stages and the different number of positive regional lymph nodes. (F, G) Kaplan‐Meier plots of the overall survival (OS) and progression‐free survival (PFS) of GC patients with high (*n* = 89) and low (*n* = 89) levels of NKX2‐1‐AS1 expression. The data shown are the mean ± SD (error bars). *P < 0.05, **P < 0.01, and ***P < 0.001. (H) Immunohistochemistry (IHC) staining of SERPINE1 in GC patients; (a) tumor tissue with negative staining (IHC 0+); (b) tumor tissue with weak staining (IHC 1+); (c) tumor tissue with weak to moderate staining (IHC 2+); (d) tumor tissue with strong staining (IHC 3+). (I) Kaplan–Meier plots of the OS of GC patients with high (*n* = 89) and low (*n* = 89) protein expression levels of SERPINE1. The data shown are the mean ± SD (error bars). *P < 0.05, **P < 0.01 and ***P < 0.001.

NKX2‐1‐AS1 expression in 178 paired tumor/nontumor tissue from GC patients was determined by qRT‐PCR. Compared with nontumor tissues, GC tumor tissues exhibited overexpression of NKX2‐1‐AS1 (*P* = 0.0001) and SERPINE1 expression (*P* = 0.0004) and downregulation of miR145‐5p expression (*P* = 0.0033; Fig. 2A–C). Further analysis of distinct clinical subgroups revealed that overexpression of NKX2‐1‐AS1 was significantly associated with advanced TNM staging (stage III‐IV) and lymph node metastases (>6 positive lymph nodes) (Fig. 2D, E). Although NKX2‐1‐AS1 expression was positively correlated with increased tumor size, higher infiltration of peritumoral tissues, peritoneum dissemination, and presence of distant metastasis, no statistically significant differences were observed among these subgroups (Additional file 2: Fig. S1A–D). Overall, the clinical data indicated that overexpression of NKX2‐1‐AS1 is associated with GC and may have a role in GC metastasis and tumor progression.

### 3.3 Association of NKX2‐1‐AS1 and SERPINE1 overexpression with GC progression and prognosis

To evaluate the prognostic value of NKX2‐1‐AS1 overexpression, we divided the expression levels in GC patients into high‐expression and low‐expression groups based on the median expression of NKX2‐1‐AS1 in GC patients. (Additional file 2: Fig. S1E). The association of NKX2‐1‐AS1 expression with the OS and progression‐free survival (PFS) in GC patients was investigated. The Kaplan‐Meier analysis revealed that patients with high expression of NKX2‐1‐AS1 in tumor tissues exhibited significantly shorter 5‐year OS (40.52% vs. 58.89%, *P* = 0.0051) and 5‐year PFS (36.67% vs. 59.93%, *P* = 0.0019), than patients with low NKX‐2‐1AS1 expression (Fig. 2F,G). Univariate analysis revealed that OS and PFS were positively correlated with T, N, and TNM staging, presence of distant metastasis, tumor tissue infiltration, peritoneal dissemination, and NKX2‐1‐AS1 overexpression (*P* < 0.01, Table 1). Multivariate analysis indicated that NKX2‐1‐AS1 was an independent predictor for OS and PFS (Table 1). Besides, TNM staging, distant metastasis, and peripheral tumor tissue infiltration were also found to be independent risk factors for OS and PFS (Table 1). To determine the association between SERPINE1 expression and patient survival, GC patients were divided into low SERPINE1 expression (0 + and 1+; *n* = 89) or high SERPINE1 expression groups (2+ and 3+; *n* = 89), based on SERPINE1 H‐score in the corresponding tumor tissue specimens (Fig. 2H). The median H‐score (0.50) was considered as the cutoff value for classifying all GC patients into high‐ and low‐expression groups (Additional file 2: Fig. S1F). Compared with patients with low SERPINE1 expression, those with high SERPINE1 expression exhibited shorter 5‐year OS (39.48% vs. 60.68%, *P* = 0.0073) (Fig. 2I). Taken together, the results suggested that NKX2‐1‐AS1 and SERPINE1 may serve as potential prognostic predictors in GC patients.

**Table 1:** Univariate and multivariate Cox regression analysis of NKX2‐1‐AS1 and survival in patients with gastric cancer. CI, confidence interval; HR, hazard ratio; TNM, tumor node metastasis.

Clinical variables	Univariate analysis		*P* value	Multivariate analysis		*P* value
	HR	95% CI		HR	95% CI	
*Overall survival*						
Gender (male vs. female)	0.736	0.498–1.196	0.276			
Age (≥60y vs. <60y)	0.949	0.621–1.448	0.936			
Tumor location (down vs. upper/middle)	0.806	0.518–1.267	0.293			
Tumor size (≥5 cm vs. <5 cm)	1.521	1.020–2.276	0.134			
Differentiation (poor vs. moderate/well)	1.845	0.937–3.745	0.071			
T stage (T3‐T4 vs. T1‐T2)	4.245	1.640–11.018	**0.005**			
N stage (N2‐N3 vs. N0‐N1)	2.317	1.287–3.546	**0.002**			
TNM stage (III‐IV vs. II‐I)	3.898	2.136–6.945	**<0.001**	2.745	1.575‐5.136	**0.004**
Distant metastasis (yes vs. no)	3.389	1.955–5.896	**<0.001**	2.054	1.201‐3.634	**0.008**
Infiltration of peritumoral tissues (yes vs. no)	2.753	1.743–3.845	**<0.001**	1.934	1.245‐3.121	**0.003**
Peritoneum dissemination (yes vs. no)	3.834	2.034–7.145	**<0.001**			
NKX2‐1‐AS1 expression (high vs. low)	0.358	0.261–0.448	**<0.001**	0.383	0.297‐0.501	**0.005**
Progression‐free survival						
Gender (male vs. female)	0.856	0.534–1.319	0.390			
Age (≥60y vs. <60y)	0.813	0.498–1.289	0.359			
Tumor location (down vs. upper/middle)	0.764	0.491–1.125	0.163			
Tumor size (≥5 cm vs. <5 cm)	1.296	0.759–1.868	0.387			
Differentiation (poor vs. moderate/well)	1.176	0.618–2.065	0.721			
T stage (T3‐T4 vs. T1‐T2)	5.780	1.792–17.265	**0.003**			
N stage (N2‐N3 vs. N0‐N1)	2.578	1.389–4.179	**0.002**			
TNM stage (III‐IV vs. II‐I)	3.619	1.895–6.191	**<0.001**	2.571	1.378–4.701	**0.002**
Distant metastasis (yes vs. no)	3.197	1.765–5.329	**<0.001**	1.921	1.102–3.397	**0.037**
Infiltration of peritumoral tissues (yes vs. no)	2.486	1.576–3.793	**<0.001**	1.846	1.138–2.878	**0.009**
Peritoneum dissemination (yes vs. no)	2.354	1.104–4.943	**0.039**			
NKX2‐1‐AS1 expression (high vs. low)	0.319	0.223–0.407	**<0.001**	0.337	0.227–0.438	**0.001**

The bold type represents *P* values smaller than 0.05.

### 3.4 NKX2‐1‐AS1 overexpression regulates GC cell proliferation, migration, and invasion

**Fig. 3:**
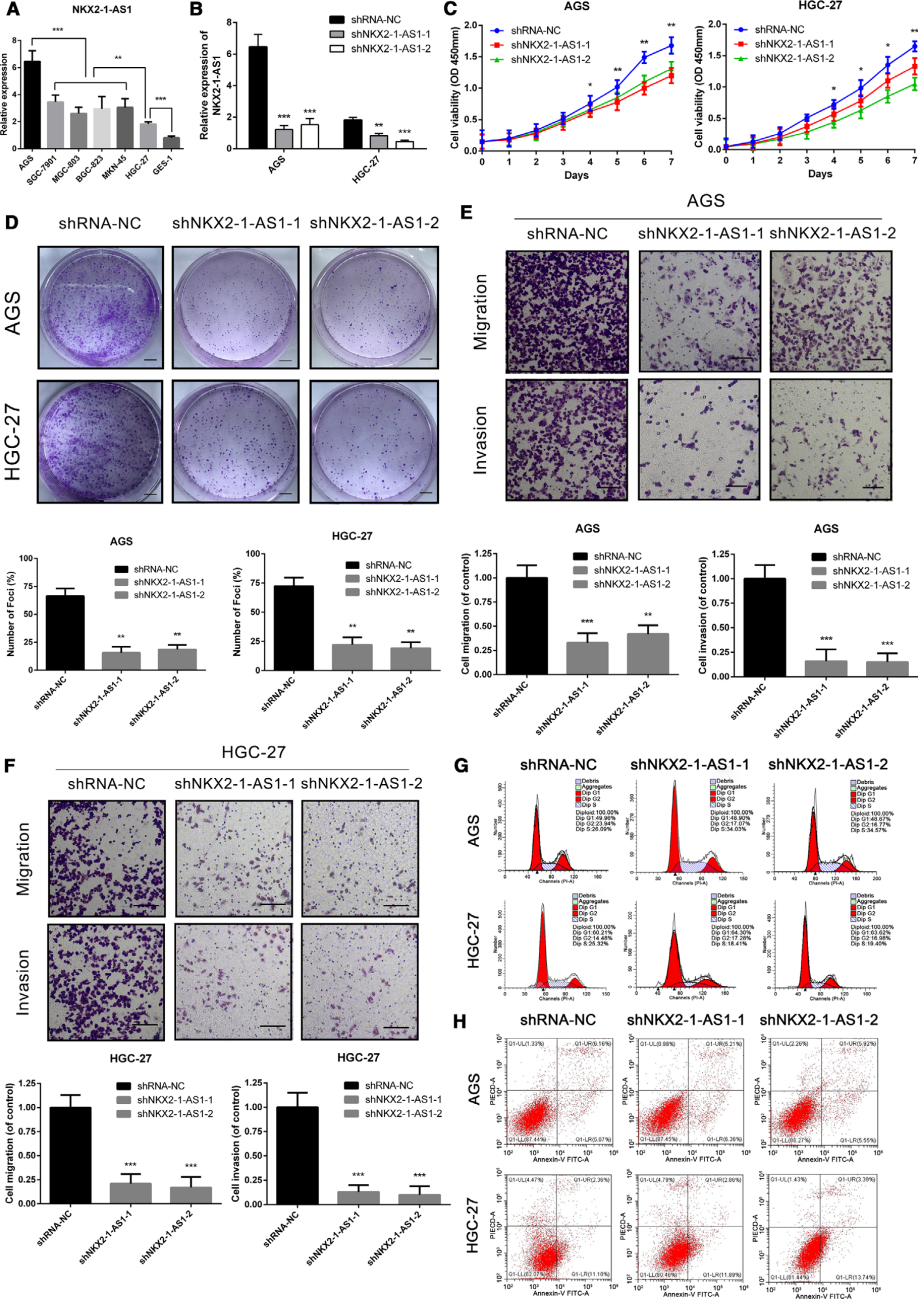
NKX2‐1‐AS1 knockdown inhibits GC cell proliferation, migration, and invasion. (A) qRT‐PCR analysis of the expression of NKX2‐1‐AS1 in six GC cell lines and the immortalized gastric normal mucosal cell line (GES‐1). (B) qRT‐PCR analysis of the expression of NKX2‐1‐AS1 in AGS and HGC‐27 cells, transfected with two independent shRNAs targeting NKX2‐1‐AS1. (C) Cell proliferation assay in AGS and HGC‐27 cells following knockdown of NKX2‐1‐AS1. (D–H) Representative images of the colony formation (scale bar = 500 µm), transwell (scale bar = 100 µm), cell cycle assay, and apoptosis assays in AGS and HGC‐27 cells following transfection with shNKX2‐1‐AS1–1 or shNKX2‐1‐AS1–2. The data shown are the mean ± SD (error bars, *n* = 3). *P < 0.05, **P < 0.01 and ***P < 0.001.

**Fig. 4:**
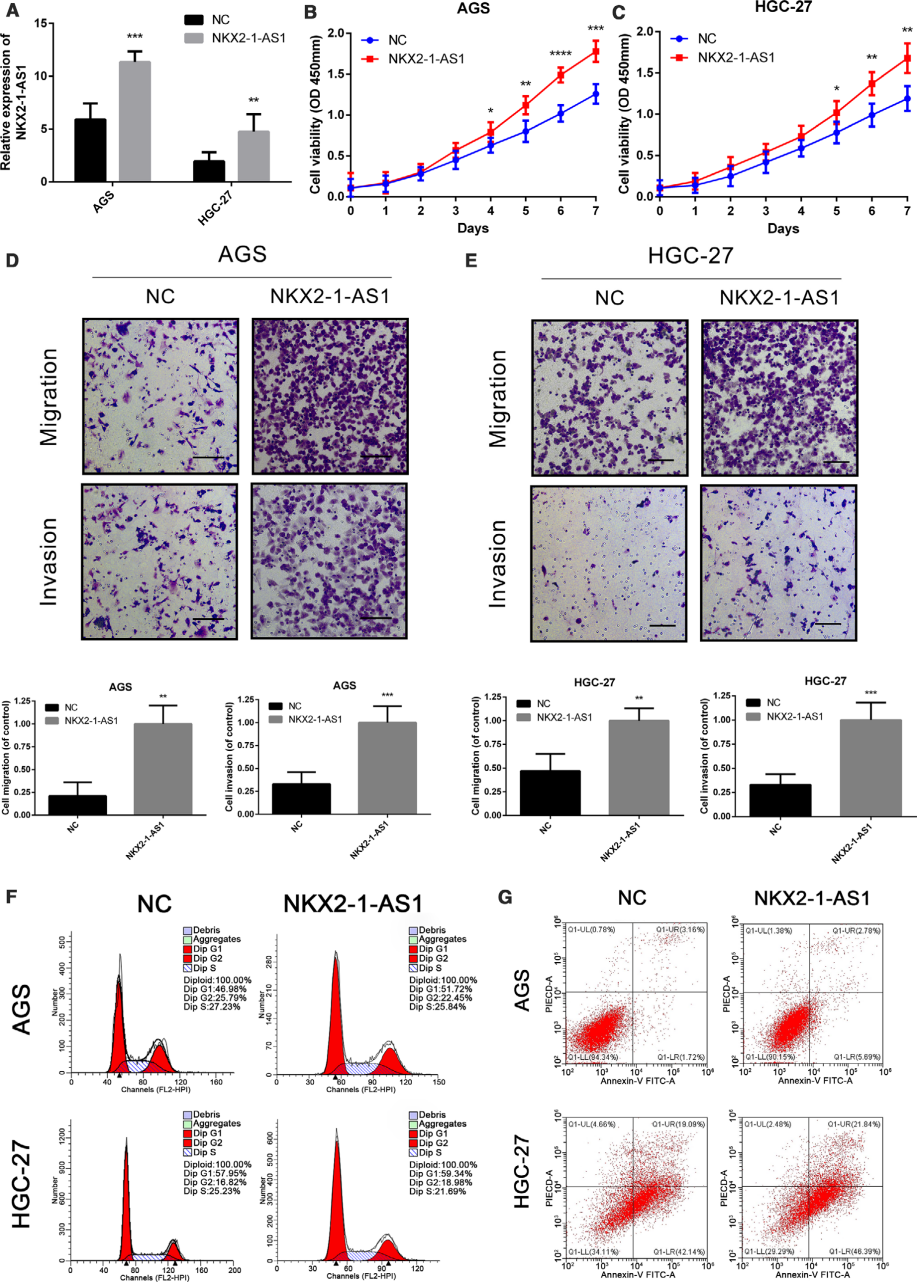
NKX2‐1‐AS1 overexpression promotes cell proliferation, migration, and invasion. (A) Expression of NKX2‐1‐AS1 is detected by qRT‐PCR in AGS and HGC‐27 cells with NKX2‐1‐AS1 overexpression. (B–E) Proliferation, migration, and invasion assays using AGS and HGC‐27 cells with NKX2‐1‐AS1 overexpression, by the cell proliferation and transwell assays (scale bars = 100 µm). F, G Representative images of cell cycle and apoptosis assays in AGS and HGC‐27 cells following NKX2‐1‐AS1 overexpression. The data shown are the mean ± SD (error bars, *n* = 3). *P < 0.05, **P < 0.01 and ***P < 0.001.

Considering that NKX2‐1‐AS1 upregulation was positively associated with GC progression and metastasis, loss‐of‐function experiments were performed to confirm the effect of NKX2‐1‐AS1 on cell proliferation, migration, and invasion of GC cells. The expression level of NKX2‐1‐AS1 in all GC cells was significantly higher than that of the immortalized gastric normal mucosal cell‐line GES‐1. AGS and HGC‐27 cells with the highest and lowest NKX2‐1‐AS1 expression, respectively, were used for further analysis (Fig. 3A). shRNA against NKX2‐1‐AS1 was used to abrogate its expression, and knockdown efficiency was assessed by qRT‐PCR (Fig. 3B). Cell count and colony formation assays indicated that NKX2‐1‐AS1 knockdown inhibited GC cell growth and colony formation (Fig. 3C,D), both of which were reversed by NKX2‐1‐AS1 overexpression (Fig. 4A–C). Furthermore, NKX2‐1‐AS1 knockdown suppressed the migratory and invasive characteristics of GC cells (Fig. 3E,F), both of which were rescued by NKX2‐1‐AS1 overexpression (Fig. 4D,E). Interestingly, there were no significant changes in the cell cycle and apoptosis rate of GC cells after NKX2‐1‐AS1 knockdown (Fig. 3G,H) or overexpression (Fig. 4F,G). Collectively, these findings suggested that NKX2‐1‐AS1 overexpression regulated GC cell growth, migration, and invasion *in vitro*; however, it had no impact on GC cell cycle and apoptosis.

### 3.5 NKX2‐1‐AS1 regulates GC cell proliferation and angiogenesis *in vivo*

**Fig. 5:**
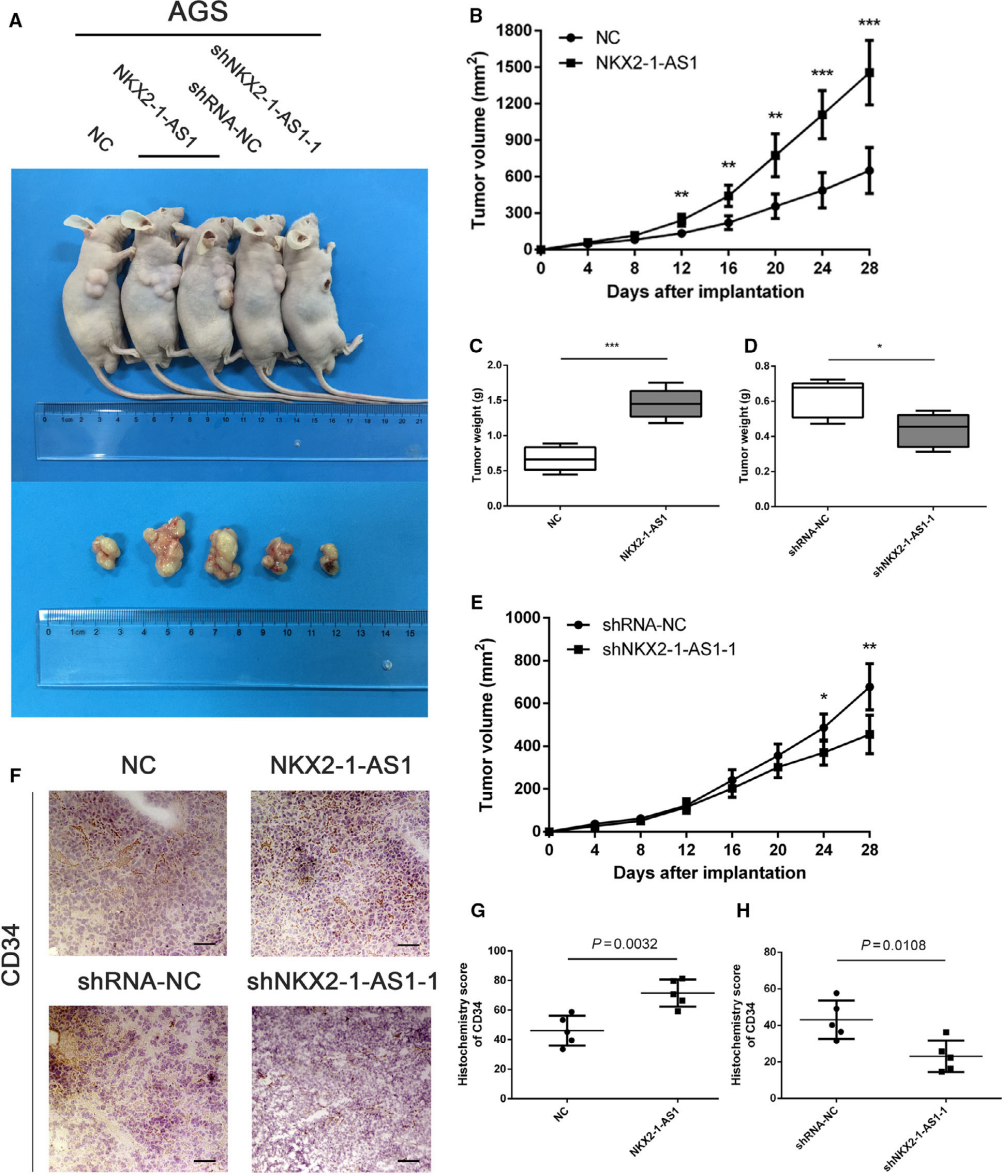
NKX2‐1‐AS1 regulates GC cell proliferation and angiogenesis *in vivo*. (A) Upper panel: AGS cells transfected with an NKX2‐1‐AS1 expression vector or empty vector and shNKX2‐1‐AS1–1 or shRNA‐NC were injected into the right armpit of BALB/c nude mice. Bottom panel: Representative images of xenograft tumors. (B, C) Volume and weight of the tumor xenograft in NKX2‐1‐AS1 overexpressing group and control group. (D, E) Weight and volume of the tumor xenograft in the NKX2‐1‐AS1 knockdown group and control group. (F) Representative IHC staining images of CD34 in corresponding xenograft specimens (scale bar = 50 µm). (G, H) Statistical analyses of H‐score of CD34 expression in corresponding xenografts. Error bars: mean ± SD from three independent experiments. The data shown are the mean ± SD (error bars). *P < 0.05, **P < 0.01 and ***P < 0.001.

To evaluate the effect of NKX2‐1‐AS1 on GC tissues *in vivo*, AGS cells with stable overexpression of NKX2‐1‐AS1 were subcutaneously injected into female nude mice (Fig. 5A). As anticipated, xenograft tumors were significantly larger and heavier in the AGS‐NKX2‐1‐AS1 group than in the negative control (NC) group (Fig. 5B,C). Similarly, NKX2‐1‐AS1 shRNA‐transfected AGS cells were also injected subcutaneously in female nude mice, and it was observed that the tumors were significantly smaller and lighter in the shNKX2‐1‐AS1–1 group as compared to the shRNA–NC group (Fig. 5D,E). During necropsy, tumors appeared paler in the shNKX2‐1‐AS1–1 group than in the shRNA–NC group; based on this observation, we hypothesized that NKX2‐1‐AS1 expression might be associated with angiogenesis in GC tissue that may affect tumor growth and progression. IHC staining in tumor specimens with NKX2‐1‐AS1 overexpression revealed enhanced CD34 expression, which was reversed by NKX2‐1‐AS1 knockdown (Fig. 5F–H). These findings strongly suggested that NKX2‐1‐AS1 was associated with enhanced growth and increased angiogenesis in GC *in vivo*.

### 3.6 NKX2‐1‐AS1 act as a ceRNA of miR‐145‐5p in GC

**Fig. 6:**
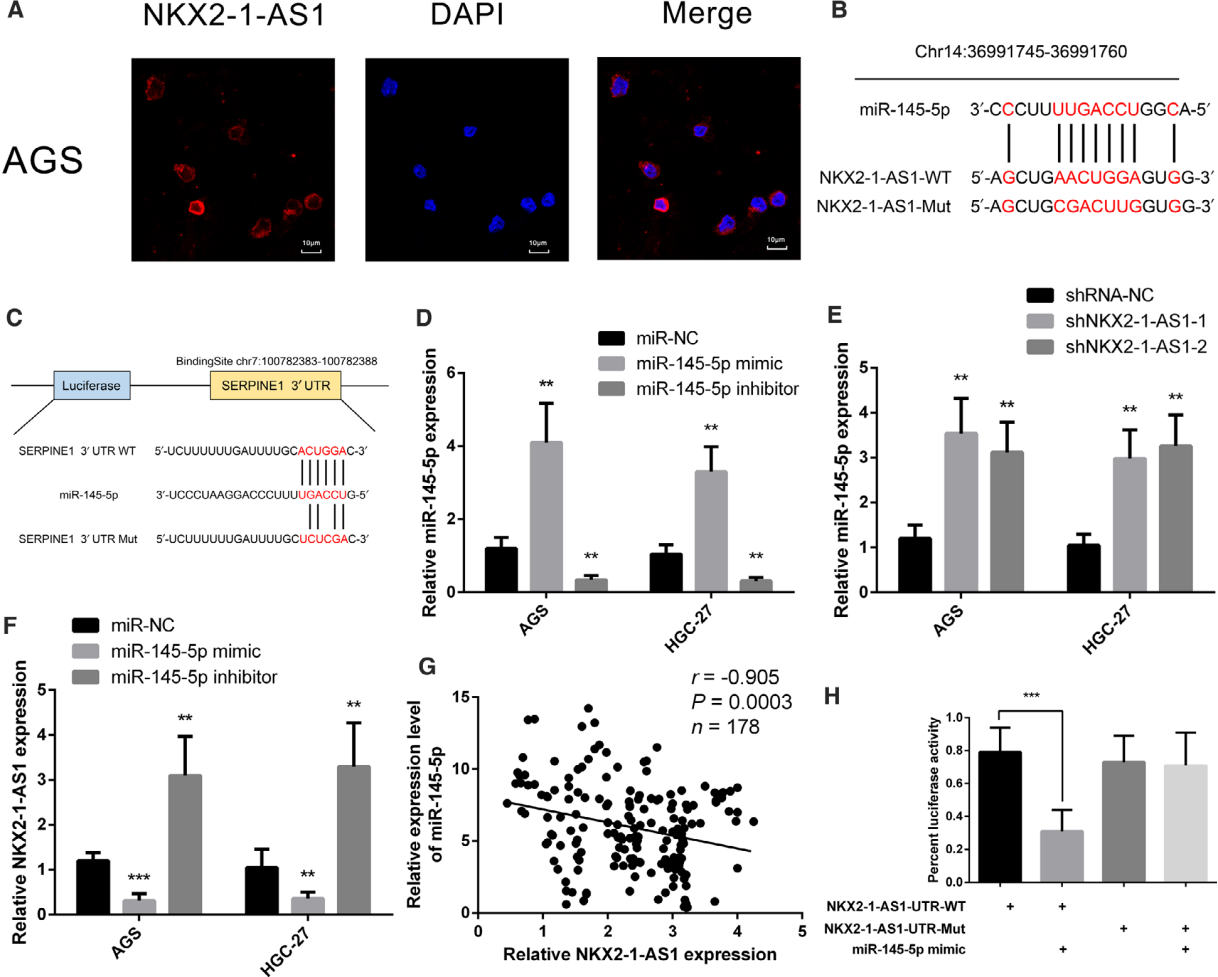
NKX2‐1‐AS1 as a ceRNA for miR‐145‐5p in GC. (A) NKX2‐1‐AS1 detection by FISH (red) was performed in AGS cells (scale bar = 10 µm). Nuclei are counterstained with DAPI (blue). (B) Schematic representation of the predicted miR‐145‐5p binding site in NKX2‐1‐AS1 using the online database Mircode Predicted algorithm. The mutated site in the 3′‐UTR of NKX2‐1‐AS1 was shown as NKX2‐1‐AS1‐Mut. The numbers indicate the nucleotides' positions in the reference wild‐type sequence of NKX2‐1‐AS1 (Ensembl version: ENSG00000253563). (C) Schematic representation of the predicted miR‐145‐5p target site within the 3’‐UTR of SERPINE1. The predicted target site for miR‐145‐5p was located at the proximal portion of the SERPINE1 3’‐UTR. Two nucleotides complementary to the seed sequence of miR‐145‐5p were mutated in the SERPINE1 mutant plasmid. The number indicates the position of the nucleotides in the reference wild‐type sequence of SERPINE1 (NM_000602.5). (D) Relative expression of miR‐145‐5p in AGS and HGC‐27 cells transfected with miR‐145‐5p mimic or inhibitor. (E) Relative expression of miR‐145‐5p in AGS and HGC‐27 cells following transfection with shNKX2‐1‐AS1 or scramble sequence. (F) Relative expression of NKX2‐1‐AS1 in AGS and HGC‐27 cells transfected with miR‐145‐5p mimic or inhibitor. (G) Correlation analysis between NKX2‐1‐AS1 and miR‐145‐5p expression in 178 GC tumor tissues. (H) Luciferase reporter assay in wild‐type (WT) and mutated (Mut) NKX2‐1‐AS1 reporter plasmid in the human embryonic kidney (HEK) 293FT cells co‐transfected with miR‐145‐5p mimic. The data shown are the mean ± SD (error bars, *n* = 3). *P < 0.05, **P < 0.01, and ***P < 0.001.

To further elucidate the regulatory mechanism of NKX2‐1‐AS1 in GC, the subcellular localization of NKX2‐1‐AS1 was determined by FISH. As illustrated in Fig. 6A, the NKX2‐1‐AS1 transcript was predominantly localized to the cytoplasm. Cytoplasmic lncRNAs are known to function as ceRNAs that attenuate the negative regulation of target mRNAs by sponging miRNAs [32]. Based on our TCGA data analysis and validation of clinical samples, we hypothesized that NKX2‐1‐AS1, miR‐145‐5p, and SERPINE1 might be involved in a regulatory ceRNA network. We predicted the binding sites between NKX2‐1‐AS1 and miR‐145‐5p (Fig. 6B) and between miR‐145‐5p and SERPINE1 (Fig. 6C) using miRcode and StarBase, respectively. To validate our hypothesis, AGS and HGC‐27 cells were transfected with miR‐145‐5p mimic or inhibitor (Fig. 6D) to up‐ and downregulate miR‐145‐5p expression, respectively. Notably, NKX2‐1‐AS1 knockdown significantly upregulated the expression of miR‐145‐5p. Conversely, under‐ and overexpression of miR‐145‐5p markedly up‐ and downregulated the expression of NKX2‐1‐AS1, respectively (Fig. 6E,F). Meanwhile, we also identified that NKX2‐1‐AS1 expression and miR‐145‐5p expression were significantly negatively correlated in GC tissues (Fig. 6G). To identify the direct evidence of interaction between NKX2‐1‐AS1 and miR‐145‐5p, NKX2‐1‐AS1 was subcloned with a wild‐type (NKX2‐1‐AS1‐WT) or mutated (NKX2‐1‐AS1‐Mut) miR‐145‐5p binding site into a dual‐luciferase reporter. As depicted in Fig. 6H, after co‐transfection with miR‐145‐5p mimic, the relative luciferase activity of NKX2‐1‐AS1‐WT in HEK293FT cells was significantly reduced, but the luciferase activity of NKX2‐1‐AS1‐Mut remained unchanged, demonstrating that miR‐145‐5p was a direct target of NKX2‐1‐AS1. Together, the data indicated that NKX2‐1‐AS1 acts as a ceRNA that regulates miR‐145‐5p.

### 3.7 NKX2‐1‐AS1 promotes angiogenesis in GC tissue by targeting miR‐145‐5p/SERPINE1

**Fig. 7:**
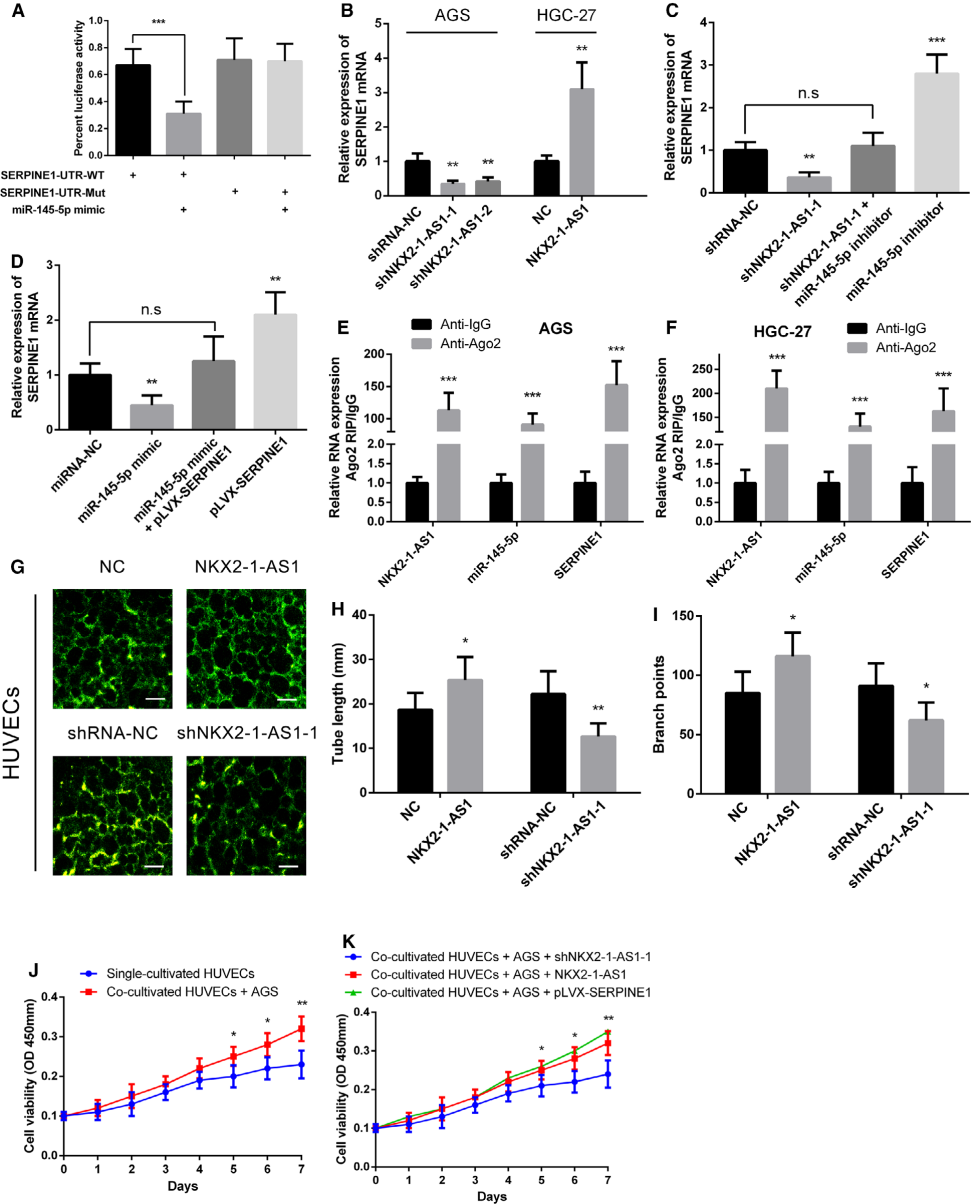
NKX2‐1‐AS1 expression promotes GC tissue angiogenesis by targeting miR‐145‐5p/SERPINE1. (A) Luciferase reporter assay in HEK‐293FT cells co‐transfected with the wild‐type (WT) or mutated (Mut) SERPINE1 3’‐UTR reporter vector and miR‐145‐5p mimic. (B) Relative expression of SERPINE1 mRNA in AGS cells with NKX2‐1‐AS1 knockdown and HGC‐27 cells with NKX2‐1‐AS1 overexpression. (C) qRT‐PCR analysis of SERPINE1 expression in AGS cells following transfection with shRNA targeting NKX2‐1‐AS1 and/or inhibition of miR‐145‐5p. (D) qRT‐PCR analysis of SERPINE1 expression in AGS cells following the ectopic expression of miR‐145‐5p and/or pLVX‐SERPINE1 expression vector lacking the 3’‐UTR. (E, F) RIP assay for detecting the binding capacity of NKX2‐1‐AS1, miR‐145‐5p, and SERPINE1 to anti‐Ago2 in AGS and HGC‐27 (anti‐IgG was used as control). (G) Tube formation assay evaluates the angiogenic capacity of HUVEC cells with overexpression or knockdown of NKX2‐1‐AS1 (scale bar = 250 µm). (H, I) Quantitative analysis of the tube length and number of branches in the tube formation assay. (J) CCK‐8 assay to detect HUVEC cell proliferation when cultured alone and co‐cultured with AGS cells. (K) NKX2‐1‐AS1 knocked down or overexpressed, or SERPINE1 overexpressed AGS cells were co‐cultured with HUVECs cells; CCK‐8 experiment was used to detect HUVECs cells proliferation activity. The data shown are the mean ± SD (error bars, *n* = 3). n.s, not significant, *P < 0.05, **P < 0.01 and ***P < 0.001.

miRNA‐mediated regulation of protein expression is accomplished by inhibiting mRNA translation or degradation of mRNAs [33]. Based on our RNAseq analysis and binding site predictions, miR‐145‐5p is predicted to bind to both SERPINE1 and MEST (Fig. 1I). Of these two upregulated mRNAs, SERPINE1 overexpression was negatively correlated with the survival of GC patients. Luciferase assay revealed that miR‐145‐5p could directly bind to complementary sequences in the 3’‐UTR of SERPINE1 (Fig. 7A). Furthermore, we observed that SERPINE1 expression was correlated with that of NKX2‐1‐AS1 (Fig. 7B). Consistently, the miR‐145‐5p inhibitor could partially rescue the inhibitory effect of NKX2‐1‐AS1 on SERPINE1 expression (Fig. 7C). Similarly, AGS cells co‐transfected with miR‐145‐5p mimic and pLVX‐SERPINE1 partially restored the expression of SERPINE1 as compared with cells transfected with miR‐145‐5p mimic only (Fig. 7D). At the same time, rescue of SERPINE1 expression in NKX2‐1‐AS1 knockdown GC cells is able to restore, or even to promote, the ability of growth, migration, invasion in GC cells (Additional file 3: Fig. S2A–C). The RIP assay revealed that NKX2‐1‐AS1, miR‐145‐5p, and SERPINE1 were significantly enriched in the anti‐Ago2 group as compared to the NC (anti‐IgG) group (Fig. 7E, F). Using the tube formation assay, we established that HUVECs with high expression of NKX2‐1‐AS1 produced a significantly increased number of vascular structures in a short period. In contrast, HUVEC cells with low expression of NKX2‐1‐AS1 exhibited significantly poor angiogenic ability compared to the NC group (Fig. 7G–I). Meanwhile, compared with HGC‐27, the expression of NKX2‐1‐AS1 in HUVECs is significantly lower, while a significantly high expression of SERPINE1 was observed in NKX2‐1‐AS1‐overexpressed HUVECs (Additional file 3: Fig. S2D, E). Furthermore, HUVECs co‐cultured with AGS exhibited enhanced proliferation compared with HUVECs in a single culture (Fig. 7J). Besides, HUVECs co‐cultured with AGS that overexpressed both NKX2‐1‐AS1 and SERPINE exhibited higher proliferative capabilities than those co‐cultured with NKX2‐1‐AS1 knockdown (Fig. 7K). Collectively, these findings demonstrated that the effect of NKX2‐1‐AS1 in GC angiogenesis was predominantly mediated through the miR‐145‐5p/SERPINE1 axis.

### 3.8 NKX2‐1‐AS1/miR‐145‐5p/SERPINE1 axis regulates GC proliferation, metastasis, invasion, and angiogenesis via activation of the VEGFR‐2 signaling pathway

**Fig. 8:**
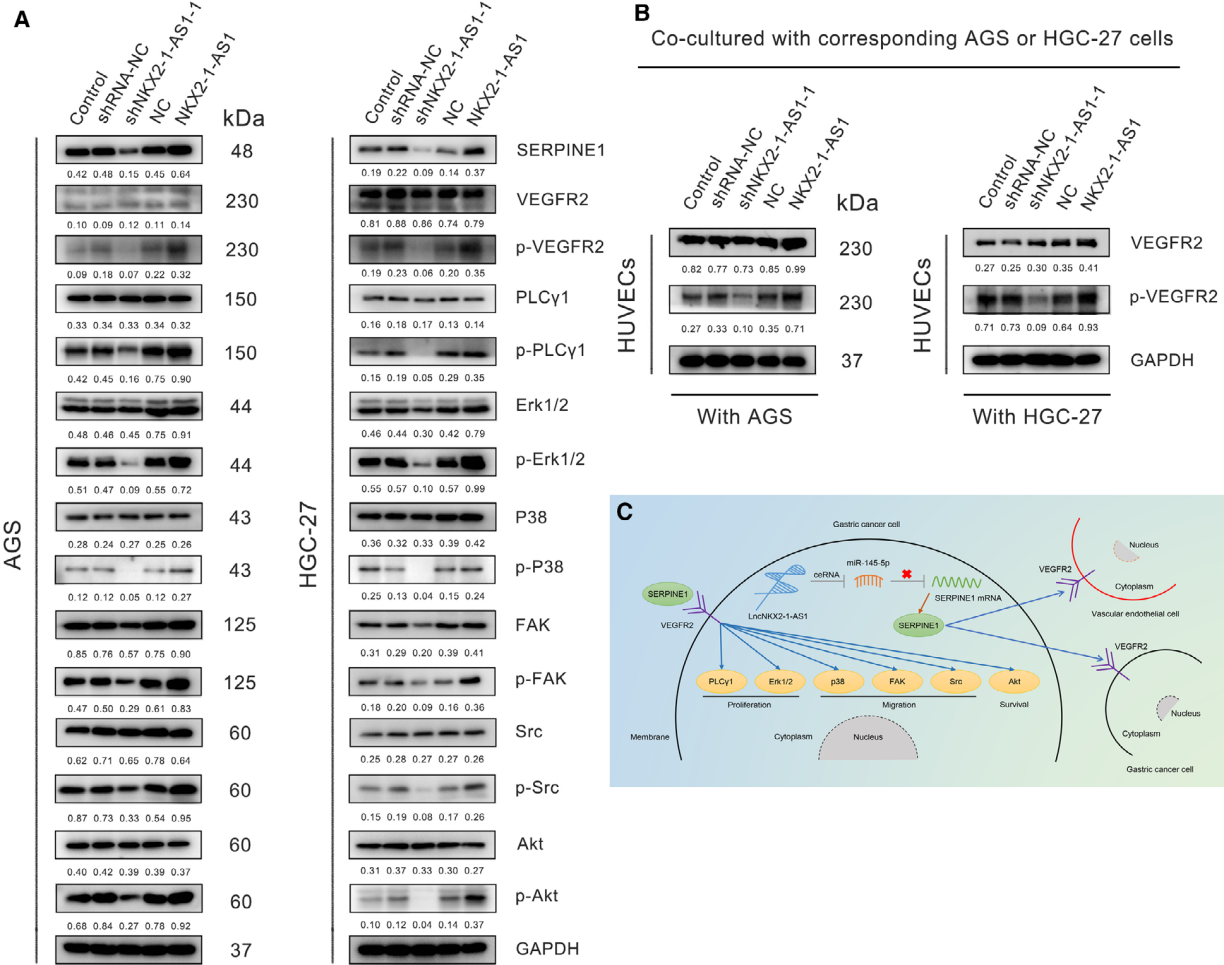
The NKX2‐1‐AS1/miR‐145‐5p/SERPINE1 axis regulates GC metastasis, invasion, and angiogenesis via activation of VEGFR‐2 signaling pathway. (A) Western blot assay was used to detect the expression of SERPINE1, VEGFR‐2, p‐VEGFR‐2, PLC‐λ, p‐PLC‐λ, Erk1/2, p‐Erk1/2, P38, p‐P38, FAK, p‐FAK, Src, p‐Src, Akt, and p‐Akt proteins in AGS and HGC‐27 cells transfected with or without shRNA‐NC, shNKX2‐1‐AS1–1, vector‐NC, or NKX2‐1‐AS1. Numbers correspond to protein quantification, using GAPDH as a loading control. (B) After HUVECs cells were co‐cultured with corresponding AGS or HGC‐27 cells with or without transfection for 7 days, the content of VEGFR‐2 and p‐VEGFR‐2 protein was determined. GAPDH serves as an internal reference. (C) Schematic representation of the regulatory mechanism associated with the NKX2‐1‐AS1/miR‐145‐5p/SERPINE1 axis in promoting GC cell proliferation, metastasis, and angiogenesis.

SERPINE1 was reported to play a crucial role in tissue angiogenesis. Thus, to determine whether NKX2‐1‐AS1/miR‐145‐5p regulated the canonical VEGFR‐2 signaling via SERPINE1, the expression and phosphorylation status of key proteins in the VEGFR‐2 signaling pathway were analyzed in GC cells following NKX2‐1‐AS1 overexpression or knockdown. Using Western blot assays, angiogenesis‐related VEGFR‐2, proliferation‐related PLCγ1 and ErK1/2, migration‐related p38, FAK, and Src, as well as survival‐related Akt proteins, were analyzed. The results revealed that NKX2‐1‐AS1 knockdown and overexpression exhibited no significant effect on total protein expression. In contrast, NKX2‐1‐AS1 knockdown caused reduced protein phosphorylation, whereas NKX2‐1‐AS1 overexpression resulted in increased protein phosphorylation, suggesting an active role of NKX2‐1‐AS1 in the regulation of VEGFR‐2 signaling (Fig. 8A). Similar findings were obtained in HUVECS co‐cultured with AGS cells (Fig. 8B). Meanwhile, rescue of SERPINE1 expression in NKX2‐1‐AS1 knockdown AGS cells is able to restore, or even to increase, the expression of p‐ERK, p‐FAK, p‐Akt, p‐Src in AGS cells (Additional file 3: Fig. S2F). Overall, these findings confirmed our hypothesis that the NKX2‐1‐AS1/miR‐145‐5p/SERPINE1 axis regulated GC cell proliferation, metastasis, invasion, and angiogenesis through the activation of the VEGFR‐2 signaling pathway.

## 4 Discussion

Dysregulation of lncRNA expression has been recently associated with GC and other gastrointestinal cancers [34]. An increasing number of recent studies have revealed the role of lncRNAs in regulating crucial cancer‐associated pathways both at transcriptional and at post‐transcriptional levels. However, for the majority of the annotated lncRNAs, the functional relevance remains enigmatic. Evidence indicates that NKX2‐1‐AS1, a lncRNA, suppresses the migration of human carcinoma cells by negatively regulating CD274/PD‐L1 and cell‐cell interaction genes [13]. In addition, the close proximity of NKX2‐1‐AS1 to the NKX2‐1 transcription factor has been associated with the progression of GC [35]. The present study indicated that NKX2‐1‐AS1 expression is significantly upregulated in GC, promoting cell proliferation and supporting angiogenesis, both *in vitro* and *in vivo*. Furthermore, *in vitro* experiments showed a positive correlation of NKX2‐1‐AS1 with metastasis and invasion potential. Taken together, the results suggested that NKX2‐1‐AS1 promotes GC progression and may serve as a potential candidate diagnostic and prognostic biomarker for GC.

Accumulating studies have suggested that the biological functions of lncRNAs are largely dependent on their distinct unique subcellular localization [36]. Cytoplasmic lncRNAs can function as mRNA decoys and modulate mRNA export, stability, localization, translation, and influence signal transduction pathways to regulate cellular metabolism [32]. Previously, the miR‐145 has been reported to inhibit GC cell migration and metastasis, likely due to the suppression of myosin VI (MYO6) expression [37]. Using bioinformatic analyses, luciferase reporter assay, and RIP assay, this study demonstrated that NKX2‐1‐AS1 could directly bind and inhibit miR‐145‐5p expression and indicated that miR‐145‐5p expression was significantly downregulated and negatively correlated with NKX2‐1‐AS1 expression in GC cells. The under‐expression of miR‐145‐5p, due to the ceRNA nature of NKX2‐1‐AS1, alleviated the downregulation of SERPINE1, thereby promoted GC progression. A similar ceRNA‐related mechanism has been reported in nasopharyngeal cancer, wherein MACC1 antisense RNA 1 (MACC1‐AS1) acts as a molecular sponge of miR‐145 [38]. Recent studies have unveiled the role of miRNAs in cancer as crucial modulators of signal transduction, leading to either translational inhibition or degradation of mRNAs [8].

It is well established that tumor angiogenesis is a key mechanism in promoting tumor growth, invasion, and metastasis [39]. An increasing number of studies indicated that miRNAs interact with several mRNA targets of the VEGF‐A pathway and regulate GC‐related angiogenesis [40]. Conversely, miR‐145 has been shown to inhibit GC cell invasion, metastasis, and angiogenesis by targeting v‐ets erythroblastosis virus E26 oncogene homolog 1 (ETS1) [41]. Moreover, inhibition of miRNA‐mediated SERPINE1 suppresses tumor angiogenesis, regardless of the angiogenic stimuli, in malignant pleural mesothelioma [42]. In the present study, we found that SERPINE1 as a direct target of miR‐145‐5p. Further analysis revealed that the loss of SERPINE1 inhibition by miR‐145‐5p significantly enhanced the activity of the VEGFR‐2 pathway. Similarly, the VEGFR‐2 pathway was significantly activated in GC cells overexpressing NKX2‐1‐AS1. Besides SERPINE1 and MEST mRNA, which exhibits potential miR‐145‐5p binding sites, were also significantly downregulated in GC on the basis of TCGA data. However, MEST overexpression was not associated with the prognosis of GC patients, and no significant differential expression was observed between tumor and nontumor tissues in the samples analyzed by this study (data not shown). Thus, we hypothesized that miR‐145‐5p regulated SERPINE1 expression in GC. By tube formation assay and co‐culture of GC cells with HUVECs, the present study provided evidence of direct interaction of NKX2‐1‐AS1 and SERPINE1 in ameliorating the proliferation of vascular endothelial cells, further confirming its crucial role in angiogenesis. During the proliferation of GC cells, we also observed that GC cells with high expression of NKX2‐1‐AS1 exhibited stronger proliferation activity. However, in cell cycle experiments, we did not observe statistically significant differences between GC cells with high NKX2‐1‐AS1 expression and the control group. This may be attributed to the fact that we counted the proportion of each cycle; however, we did not evaluate the overall cycle rate. In future research, we will consider including the cell cycle rate to explain this concern better. Taken together, the findings of the present study indicated that the NKX2‐1‐AS1/miR‐145‐5p axis activated the VEGFR‐2 signaling pathways via the upregulation of SERPINE1 expression in GC.

VEGFR‐2 is mainly expressed by both vascular endothelial and GC cells. Conversely, SERPINE1 proteins are mainly found in the cytoplasm and are directly associated with extracellular matrix remodeling. This suggests that SERPINE1 proteins are transported out of GC cells, possibly via exosomes, and act on the tumor microenvironment to promote GC tissue angiogenesis and enhance GC cell proliferation and metastasis (Fig. 8C). Collectively, the present study highlighted the significance of NKX2‐1‐AS1 as a ceRNA for miR‐145‐5p to upregulate SERPINE1, leading to increased activation of the VEGFR‐2 signaling pathway, thereby promoting tumor angiogenesis, proliferation, and metastasis in GC. However, the exact drivers of SERPINE1 localization remain to be elucidated; thus, further mechanistic studies underlying SERPINE1‐VEGFR‐2 interaction are warranted further to clarify the implication of this interaction in GC progression.

## 5 Conclusion

In summary, this study revealed that NKX2‐1‐AS1 upregulation was frequently associated with GC and might serve as an independent prognostic biomarker in GC. Furthermore, NKX2‐1‐AS1 promoted cell proliferation and tumor angiogenesis in GC both *in vitro* and *in vivo*. The present study also highlighted the molecular mechanisms by which NKX2‐1‐AS1 directly targets miR‐145‐5p to upregulate SERPINE1 and activate the VEGFR‐2 signaling pathways that promoted tumor progression and angiogenesis in GC cells (Fig. 8C). Collectively, this study suggested that NKX2‐1‐AS1 might serve as a new prognostic indicator and a potential therapeutic target for GC.

## 6 Ethics approval and consent to participate

This study was approved by the Human Research Ethics Review Board of Minhang Hospital, Fudan University. Written informed consent was obtained from all enrolled patients, and all relevant investigations were performed according to the principles of the Declaration of Helsinki. All animal studies were performed with approval from the Institutional Animal Care and Use Committee of Fudan University.

## Consent for publication

Consent to publish has been obtained from all authors.

## Conflict of interests

The authors declare no conflict of interest.

## Authors' contributions

FT and JXZ contributed equally to this study. FT, YC, XDS, YJG, and PHW performed all experiments. ML, SQL, and YOC collected tissue samples and the clinical data. JXZ, CS, and CCS analyzed and interpreted the data; and FT, JXZ, and SQL drafted the manuscript. All authors read and approved the final manuscript.

### Peer Review

The peer review history for this article is available at https://publons.com/publon/10.1002/1878‐0261.12911.

## Supporting information


**Table S1.** Primer and oligonucleotide sequences used in this study.
**Fig. S1.** Expression of NKX2‐1‐AS1 in GC patients from different clinical characteristics.
**Fig. S2.** The rescue of SERPINE1 expression in NKX2‐1‐AS1 knockdown GC cells is able to restore the function of cell growth, migration, invasion and activate VEGFR‐2 signaling pathway.
**Fig. S3.** The rescue of SERPINE1 expression in NKX2‐1‐AS1 knockdown GC cells is able to restore the function of cell growth, migration, invasion and activate VEGFR‐2 signaling pathway.

## Data Availability

All data in our study are available upon request.

## References

[1] GBD 2017 Stomach Cancer Collaborators . (2020) The global, regional, and national burden of stomach cancer in 195 countries, 1990–2017: a systematic analysis for the Global Burden of Disease study 2017. Lancet Gastroenterol Hepatol 5, 42–54. 31648970 10.1016/S2468-1253(19)30328-0 PMC7033564

[2] Torre LA , Bray F , Siegel RL , Ferlay J , Lortet‐Tieulent J & Jemal A . (2015) Global cancer statistics, 2012. CA Cancer J Clin 65, 87–108. 25651787 10.3322/caac.21262

[3] Van Cutsem E , Sagaert X , Topal B , Haustermans K & Prenen H (2016) Gastric cancer. Lancet (London, England) 388, 2654–2664. 10.1016/S0140-6736(16)30354-3 27156933

[4] Catalano V , Labianca R , Beretta GD , Gatta G , de Braud F & Van Cutsem E (2009) Gastric cancer. Crit Rev Oncol/Hematol 71, 127–164. 10.1016/j.critrevonc.2009.01.004 19230702

[5] Deng M , Zeng C , Lu X , He X , Zhang R , Qiu Q , Zheng G , Jia X , Liu H & He Z (2017) miR‐218 suppresses gastric cancer cell cycle progression through the CDK6/Cyclin D1/E2F1 axis in a feedback loop. Cancer Lett 403, 175–185. 28634044 10.1016/j.canlet.2017.06.006

[6] Nienhüser H & Schmidt T (2017) Angiogenesis and anti‐angiogenic therapy in gastric cancer. Int J Mol Sci 19: 43. 10.3390/ijms19010043 PMC5795993 29295534

[7] Chen M , Wu X , Ma W , Zhou Q , Wang X , Zhang R , Wang J & Yang X (2017) Decreased expression of lncRNA VPS9D1‐AS1 in gastric cancer and its clinical significance. Cancer Biomark 21, 23–28. 29036784 10.3233/CBM-170172 PMC13075736

[8] Bracken CP , Scott HS & Goodall GJ (2016) A network‐biology perspective of microRNA function and dysfunction in cancer. Nat Rev Genetics 17, 719–732. 27795564 10.1038/nrg.2016.134

[9] Xue X , Yang YA , Zhang A , Fong KW , Kim J , Song B , Li S , Zhao JC & Yu J (2016) LncRNA HOTAIR enhances ER signaling and confers tamoxifen resistance in breast cancer. Oncogene 35, 2746–2755. 26364613 10.1038/onc.2015.340 PMC4791209

[10] Kong J , Sun W , Li C , Wan L , Wang S , Wu Y , Xu E , Zhang H & Lai M (2016) Long non‐coding RNA LINC01133 inhibits epithelial‐mesenchymal transition and metastasis in colorectal cancer by interacting with SRSF6. Cancer Lett 380, 476–484. 27443606 10.1016/j.canlet.2016.07.015

[11] Li Z , Wu X , Gu L , Shen Q , Luo W , Deng C , Zhou Q , Chen X , Li Y , Lim Z *et al*, (2017) Long non‐coding RNA ATB promotes malignancy of esophageal squamous cell carcinoma by regulating miR‐200b/Kindlin‐2 axis. Cell Death Dis 8, e2888. 28640252 10.1038/cddis.2017.245 PMC5520904

[12] Ma L , Zhou Y , Luo X , Gao H , Deng X & Jiang Y (2017) Long non‐coding RNA XIST promotes cell growth and invasion through regulating miR‐497/MACC1 axis in gastric cancer. Oncotarget 8, 4125–4135. 27911852 10.18632/oncotarget.13670 PMC5354817

[13] Kathuria H , Millien G , McNally L , Gower AC , Tagne JB , Cao Y & Ramirez MI (2018) NKX2‐1‐AS1 negatively regulates CD274/PD‐L1, cell‐cell interaction genes, and limits human lung carcinoma cell migration. Sci Rep 8, 14418. 30258080 10.1038/s41598-018-32793-5 PMC6158174

[14] Wang J , Ding Y , Wu Y & Wang X (2020) Identification of the complex regulatory relationships related to gastric cancer from lncRNA‐miRNA‐mRNA network. J Cell Biochem 121, 876–887. 31452262 10.1002/jcb.29332

[15] Binder BR , Mihaly J & Prager GW (2007) uPAR‐uPA‐PAI‐1 interactions and signaling: a vascular biologist's view. Thromb Haemost 97, 336–342. 17334498

[16] Rakic JM , Maillard C , Jost M , Bajou K , Masson V , Devy L , Lambert V , Foidart JM & Noël A (2003) Role of plasminogen activator‐plasmin system in tumor angiogenesis. Cell Mol Life Sci 60, 463–473. 12737307 10.1007/s000180300039 PMC11138537

[17] Liao P , Li W , Liu R , Teer JK , Xu B , Zhang W , Li X , McLeod HL & He Y (2018) Genome‐scale analysis identifies SERPINE1 and SPARC as diagnostic and prognostic biomarkers in gastric cancer. OncoTargets Ther 11, 6969–6980. 10.2147/OTT.S173934 PMC6199229 30410354

[18] Yang JD , Ma L & Zhu Z (2019) SERPINE1 as a cancer‐promoting gene in gastric adenocarcinoma: facilitates tumour cell proliferation, migration, and invasion by regulating EMT. J Chemother (Florence, Italy) 31, 408–418. 10.1080/1120009X.2019.1687996 31724495

[19] Bajou K , Maillard C , Jost M , Lijnen RH , Gils A , Declerck P , Carmeliet P , Foidart JM & Noel A (2004) Host‐derived plasminogen activator inhibitor‐1 (PAI‐1) concentration is critical for in vivo tumoral angiogenesis and growth. Oncogene 23, 6986–6990. 15286708 10.1038/sj.onc.1207859

[20] Bajou K , Noël A , Gerard RD , Masson V , Brunner N , Holst‐Hansen C , Skobe M , Fusenig NE , Carmeliet P , Collen D & *et al*, (1998) Absence of host plasminogen activator inhibitor 1 prevents cancer invasion and vascularization. Nat Med 4, 923–928. 9701244 10.1038/nm0898-923

[21] Gutierrez LS , Schulman A , Brito‐Robinson T , Noria F , Ploplis VA & Castellino FJ (2000) Tumor development is retarded in mice lacking the gene for urokinase‐type plasminogen activator or its inhibitor, plasminogen activator inhibitor‐1. Can Res 60, 5839–5847. 11059781

[22] Bajou K , Masson V , Gerard RD , Schmitt PM , Albert V , Praus M , Lund LR , Frandsen TL , Brunner N , Dano K *et al*, (2001) The plasminogen activator inhibitor PAI‐1 controls in vivo tumor vascularization by interaction with proteases, not vitronectin. Implications for antiangiogenic strategies. J Cell Biol 152, 777–784. 11266468 10.1083/jcb.152.4.777 PMC2195770

[23] Loskutoff DJ , Curriden SA , Hu G & Deng G (1999) Regulation of cell adhesion by PAI‐1. APMIS 107, 54–61. 10190280 10.1111/j.1699-0463.1999.tb01526.x

[24] Waltz DA , Natkin LR , Fujita RM , Wei Y & Chapman HA (1997) Plasmin and plasminogen activator inhibitor type 1 promote cellular motility by regulating the interaction between the urokinase receptor and vitronectin. J Clin Investig 100, 58–67. 9202057 10.1172/JCI119521 PMC508165

[25] Bajou K , Peng H , Laug WE , Maillard C , Noel A , Foidart JM , Martial JA & DeClerck YA (2008) Plasminogen activator inhibitor‐1 protects endothelial cells from FasL‐mediated apoptosis. Cancer Cell 14, 324–334. 18835034 10.1016/j.ccr.2008.08.012 PMC2630529

[26] Masuda T , Hattori N , Senoo T , Akita S , Ishikawa N , Fujitaka K , Haruta Y , Murai H & Kohno N (2013) SK‐216, an inhibitor of plasminogen activator inhibitor‐1, limits tumor progression and angiogenesis. Mol Cancer Ther 12, 2378–2388. 23990114 10.1158/1535-7163.MCT-13-0041

[27] Robinson MD , McCarthy DJ & Smyth GK (2010) edgeR: a Bioconductor package for differential expression analysis of digital gene expression data. Bioinformatics (Oxford, England) 26, 139–140. 10.1093/bioinformatics/btp616 PMC2796818 19910308

[28] Ito K & Murphy D (2013) Application of ggplot2 to Pharmacometric Graphics. CPT: Pharm Syst Pharmacol 2, e79. 10.1038/psp.2013.56 PMC3817376 24132163

[29] Shannon P , Markiel A , Ozier O , Baliga NS , Wang JT , Ramage D , Amin N , Schwikowski B & Ideker T (2003) Cytoscape: a software environment for integrated models of biomolecular interaction networks. Gen Res 13, 2498–2504. 10.1101/gr.1239303 PMC403769 14597658

[30] Yeo W , Chan SL , Mo FK , Chu CM , Hui JW , Tong JH , Chan AW , Koh J , Hui EP , Loong H *et al*, (2015) Phase I/II study of temsirolimus for patients with unresectable Hepatocellular Carcinoma (HCC)‐ a correlative study to explore potential biomarkers for response. BMC Cancer 15, 395. 25962426 10.1186/s12885-015-1334-6 PMC4434865

[31] Gonzalez‐King H , García NA , Ontoria‐Oviedo I , Ciria M , Montero JA & Sepúlveda P (2017) Hypoxia Inducible Factor‐1α Potentiates Jagged 1‐Mediated Angiogenesis by Mesenchymal Stem Cell‐Derived Exosomes. Stem Cells (Dayton, Ohio) 35, 1747–1759. 10.1002/stem.2618 28376567

[32] Schmitt AM & Chang HY (2016) Long noncoding RNAs in cancer pathways. Cancer Cell 29, 452–463. 27070700 10.1016/j.ccell.2016.03.010 PMC4831138

[33] Sandler A , Gray R , Perry MC , Brahmer J , Schiller JH , Dowlati A , Lilenbaum R & Johnson DH (2006) Paclitaxel‐carboplatin alone or with bevacizumab for non‐small‐cell lung cancer. New Engl J Med 355, 2542–2550. 17167137 10.1056/NEJMoa061884

[34] Yang XZ , Cheng TT , He QJ , Lei ZY , Chi J , Tang Z , Liao QX , Zhang H , Zeng LS & Cui SZ (2018) LINC01133 as ceRNA inhibits gastric cancer progression by sponging miR‐106a‐3p to regulate APC expression and the Wnt/β‐catenin pathway. Mol Cancer 17, 126. 30134915 10.1186/s12943-018-0874-1 PMC6106894

[35] Zhao BW , Jiang SS , Chen YM , Huang CY & Li YF (2014) Reduced NKX2.1 expression predicts poor prognosis of gastric carcinoma. PLoS One 9, e114556. 25478793 10.1371/journal.pone.0114556 PMC4257675

[36] Chen LL (2016) Linking Long Noncoding RNA Localization and Function. Trends Biochem Sci 41, 761–772. 27499234 10.1016/j.tibs.2016.07.003

[37] Lei C , Du F , Sun L , Li T , Li T , Min Y , Nie A , Wang X , Geng L , Lu Y *et al*, (2017) miR‐143 and miR‐145 inhibit gastric cancer cell migration and metastasis by suppressing MYO6. Cell Death Dis 8, e3101. 29022908 10.1038/cddis.2017.493 PMC5682659

[38] Chen S , Luo X , Wu W , Li Y , Yu H , Wang Y & Yan J (2020) The long non‐coding RNA MACC1‐AS1 promotes nasopharyngeal carcinoma cell stemness via suppressing miR‐145‐mediated inhibition on SMAD2/MACC1‐AS1 axis. Biomed Pharmacoth 125, 109986. 10.1016/j.biopha.2020.109986 32058221

[39] Sammarco G , Varricchi G , Ferraro V , Ammendola M , De Fazio M , Altomare DF , Luposella M , Maltese L , Currò G , Marone G *et al*, (2019) Mast Cells, Angiogenesis and Lymphangiogenesis in Human Gastric Cancer. Int J Mol Sci 20, 2106. 10.3390/ijms20092106 PMC6540185 31035644

[40] Zhang X , Tang J , Zhi X , Xie K , Wang W , Li Z , Zhu Y , Yang L , Xu H & Xu Z (2017) Correction: miR‐874 functions as a tumor suppressor by inhibiting angiogenesis through STAT3/VEGF‐A pathway in gastric cancer. Oncotarget 8, 29535. 28468128 10.18632/oncotarget.17402 PMC5438749

[41] Zheng L , Pu J , Qi T , Qi M , Li D , Xiang X , Huang K & Tong Q (2013) miRNA‐145 targets v‐ets erythroblastosis virus E26 oncogene homolog 1 to suppress the invasion, metastasis, and angiogenesis of gastric cancer cells. Mol Cancer Res 11, 182–193. 23233482 10.1158/1541-7786.MCR-12-0534

[42] Takayama Y , Hattori N , Hamada H , Masuda T , Omori K , Akita S , Iwamoto H , Fujitaka K & Kohno N (2016) Inhibition of PAI‐1 limits tumor angiogenesis regardless of angiogenic stimuli in malignant pleural mesothelioma. Can Res 76, 3285–3294. 10.1158/0008-5472.CAN-15-1796 27197170

