# Supplementary material for: LncRNA NKX2‐1‐AS1 promotes tumor progression and angiogenesis via upregulation of SERPINE1 expression and activation of the VEGFR‐2 signaling pathway in gastric cancer
Source: Mol Oncol. 2021 Feb 13;15(4):1234–55. doi: 10.1002/1878-0261.12911 (PMC8024734; doi:10.1002/1878-0261.12911)
Supplement: Supplementary file 1 — Table S1. Primer and oligonucleotide sequences used in this study. Fig. S1. Expression of NKX2‐1‐AS1 in GC patients from different clinical characteristics. Fig. S2. The rescue of SERPINE1 expression in NKX2‐1‐AS1 knockdown GC cells is able to restore the function of cell growth, migration, invasion and activate VEGFR‐2 signaling pathway. Fig. S3. The rescue of SERPINE1 expression in NKX2‐1‐AS1 knockdown GC cells is able to restore the function of cell growth, migration, invasion and activate VEGFR‐2 signaling pathway. [file MOL2-15-1234-s001.pdf]

Table S1. Primers and oligonucleotides sequences used in this study

Name	Sequence 5'-3'
NKX2-1-AS1-forward	ACGCCGATCTTGTTGGATGT
NKX2-1-AS1-reverse	CCTCTGGTGGCTGCCTAAAA
NKX2-1-AS1-shRNA-1-forward	CCGGGCGTAAGCGCTAAAGCAACAACTCGAGT TGTTGCTTTAGCGCTTACGCTTTTTG
NKX2-1-AS1-shRNA-1-reverse	AATTCAAAAAGCGTAAGCGCTAAAGCAACAAC TCGAGTTGTTGCTTTAGCGCTTACGC
NKX2-1-AS1-shRNA-2-forward	CCGGTAGAAGCCTACATCTTGCCCGCTCGAGCG GGCAAGATGTAGGCTTCTATTTTTG
NKX2-1-AS1-shRNA-2-reverse	AATTCAAAAATAGAAGCCTACATCTTGCCCGCT CGAGCGGGCAAGATGTAGGCTTCTA
NKX2-1-AS1-FISH probe	GGAGTCGTGTGCTTTGGACT
SERPINE1-forward	GCAACGTGGTTTTCTCACCC
SERPINE1-reverse	CTCTAGGGGCTTCCTGAGGT
miR-145-5p-forward	CTCACGGTCCAGTTTTCCCA
miR-145-5p -reverse	ACCTCAAGAACAGTATTTCCAGG
U6 snRNA-forward	CTCGCTTCGGCAGCACA
U6 snRNA-reverse	AACGCTTCACGAATTTGCGT
GAPDH-forward	AATGGGCAGCCGTTAGGAAA
GAPDH-reverse	GCGCCCAATACGACCAAATC

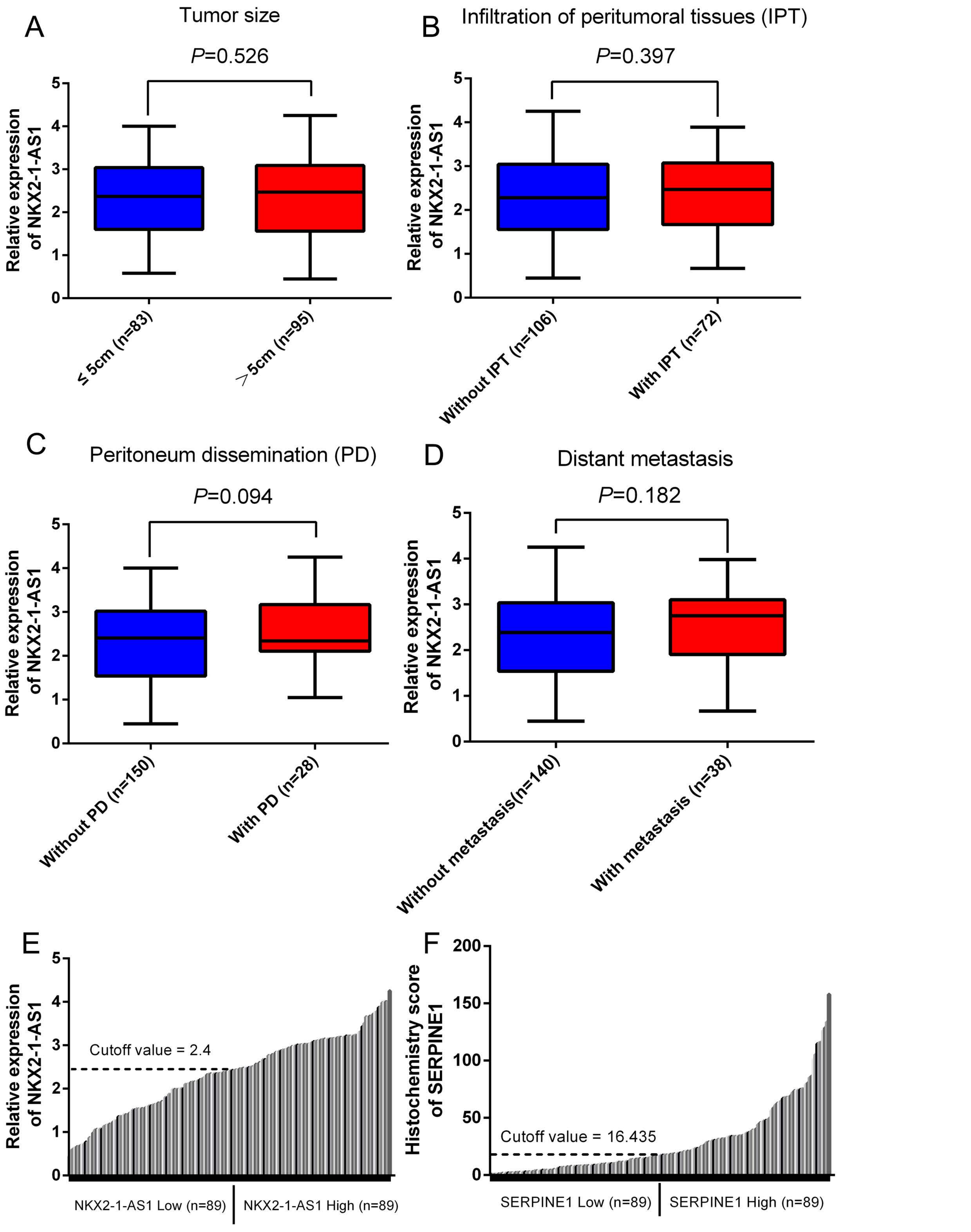
A
Tumor size
P=0.526
Relative expression of NKX2-1-AS1
≤ 5cm (n=83)
> 5cm (n=95)
B
Infiltration of peritumoral tissues (IPT)
P=0.397
Relative expression of NKX2-1-AS1
Without IPT (n=106)
With IPT (n=72)
C
Peritoneum dissemination (PD)
P=0.094
Relative expression of NKX2-1-AS1
Without PD (n=150)
With PD (n=28)
D
Distant metastasis
P=0.182
Relative expression of NKX2-1-AS1
Without metastasis(n=140)
With metastasis (n=38)
E
Relative expression of NKX2-1-AS1
Cutoff value = 2.4
NKX2-1-AS1 Low (n=89)
NKX2-1-AS1 High (n=89)
F
Histochemistry score of SERPINE1
Cutoff value = 16.435
SERPINE1 Low (n=89)
SERPINE1 High (n=89)

Figure S1. Expression of NKX2-1-AS1 in GC patients from different clinical characteristics. A-D Relative expression of NKX2-1-AS1 in GC patients with different tumor sizes, infiltration of peritumoral tissues (IPT), peritoneum dissemination (PD), and presence of distant metastasis. Results presented as the relative expression in tumor tissues relative to normal tissues. **E** qRT-PCR analysis of NKX2-1-AS1 expression in 178 GC tissues. The expression of NKX2-1-AS1 presented as the relative expression in tumor tissue to that of matched normal tissues. GC patients were divided into high NKX2-1-AS1 expression (n = 89) and low NKX2-1-AS1 expression (n = 89) groups based on the median value (0.50). **F** H-score of SERPINE1 expression in 178 GC cancer tissues. GC patients were divided into high SERPINE1 expression (n = 89) and low SERPINE1 expression (n = 89) groups based on the median value (0.50). The data shown are the *mean* ± *SD* (*error bars*). *P < 0.05, **P < 0.01 and ***P < 0.001.

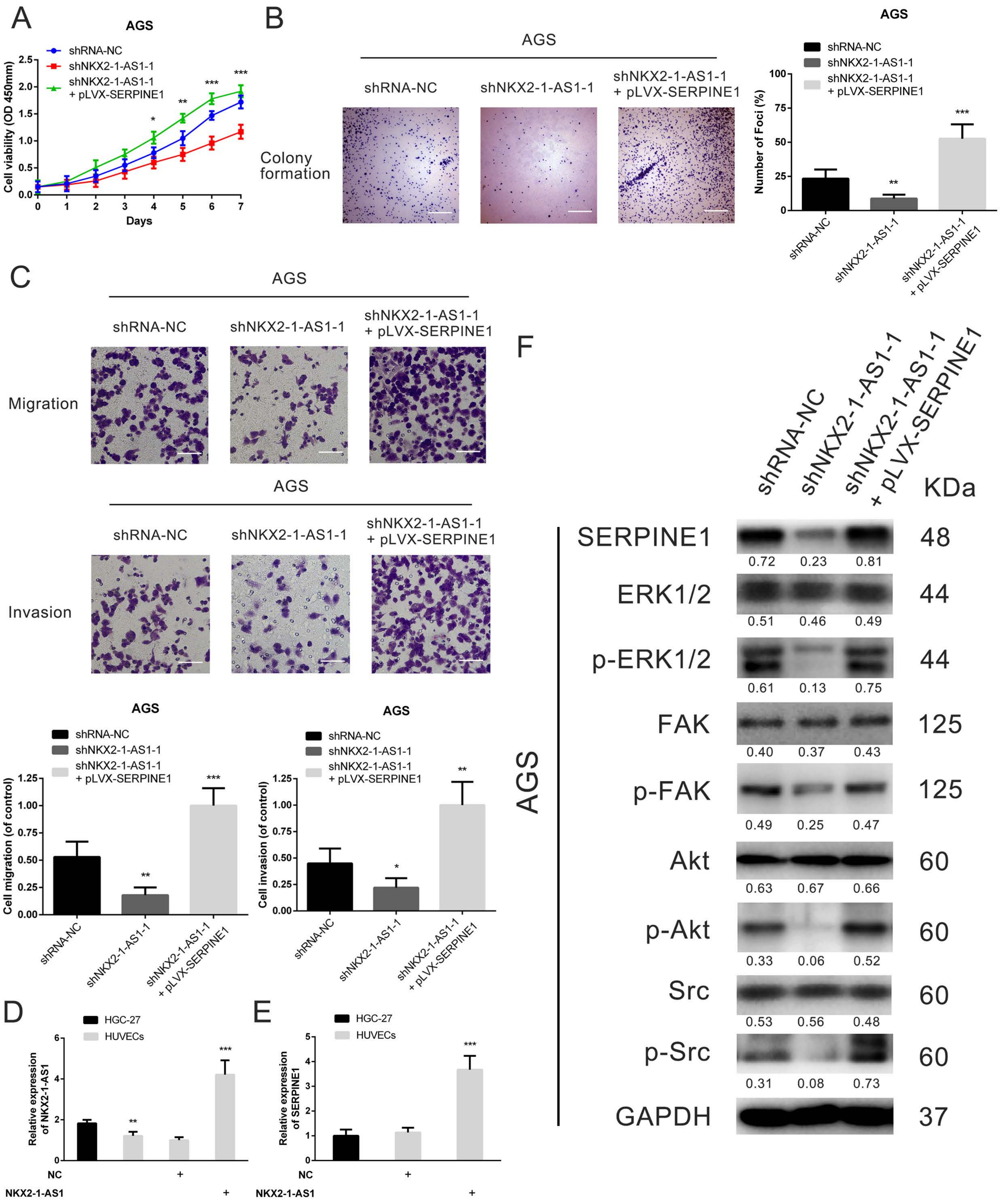
A
AGS
shRNA-NC
shNKX2-1-AS1-1
shNKX2-1-AS1-1
+ pLVX-SERPINE1
Cell viability (OD 450mm)
Days
B
AGS
shRNA-NC
shNKX2-1-AS1-1
shNKX2-1-AS1-1
+ pLVX-SERPINE1
Colony formation
Number of Foci (%)
C
AGS
Migration
Invasion
Cell migration (of control)
Cell invasion (of control)
D
HGC-27
HUVECs
Relative expression of NKX2-1-AS1
NC
NKX2-1-AS1
E
Relative expression of SERPINE1
F
KDa
SERPINE1 0.72 0.23 0.81 48
ERK1/2 0.51 0.46 0.49 44
p-ERK1/2 0.61 0.13 0.75 44
FAK 0.40 0.37 0.43 125
p-FAK 0.49 0.25 0.47 125
Akt 0.63 0.67 0.66 60
p-Akt 0.33 0.06 0.52 60
Src 0.53 0.56 0.48 60
p-Src 0.31 0.08 0.73 60
GAPDH 37

Figure S2. The rescue of SERPINE1 expression in NKX2-1-AS1 knockdown GC cells is able to restore the function of cell growth, migration, invasion and activate VEGFR-2 signaling pathway. A Cell proliferation assay in NKX2-1-AS1 knockdown AGS cells following rescue of SERPINE1 expression. **B-C** Representative images of the colony formation (scale bar = 500 μm), transwell (scale bar = 50 μm) in NKX2-1-AS1 knockdown AGS cells following rescue of SERPINE1 expression. **D-E** The NKX2-1-AS1 expression in HUVECs compared to HGC-27 cells. The relative expression of NKX2-1-AS1 and SERPINE1 in HUVECs with or without overexpression of NKX2-1-AS1. **F** Western blot assay was used to detect the expression of SERPINE1, Erk1/2, p-Erk1/2, FAK, p-FAK, Akt, p-Akt, Src and p-Src proteins in NKX2-1-AS1 knockdown AGS cells following rescue of SERPINE1 expression. Numbers correspond to protein quantification, using GAPDH as a loading control. The data shown are the *mean* ± *SD* (*error bars*). $^*P < 0.05$, $^{**}P < 0.01$ and $^{***}P < 0.001$.

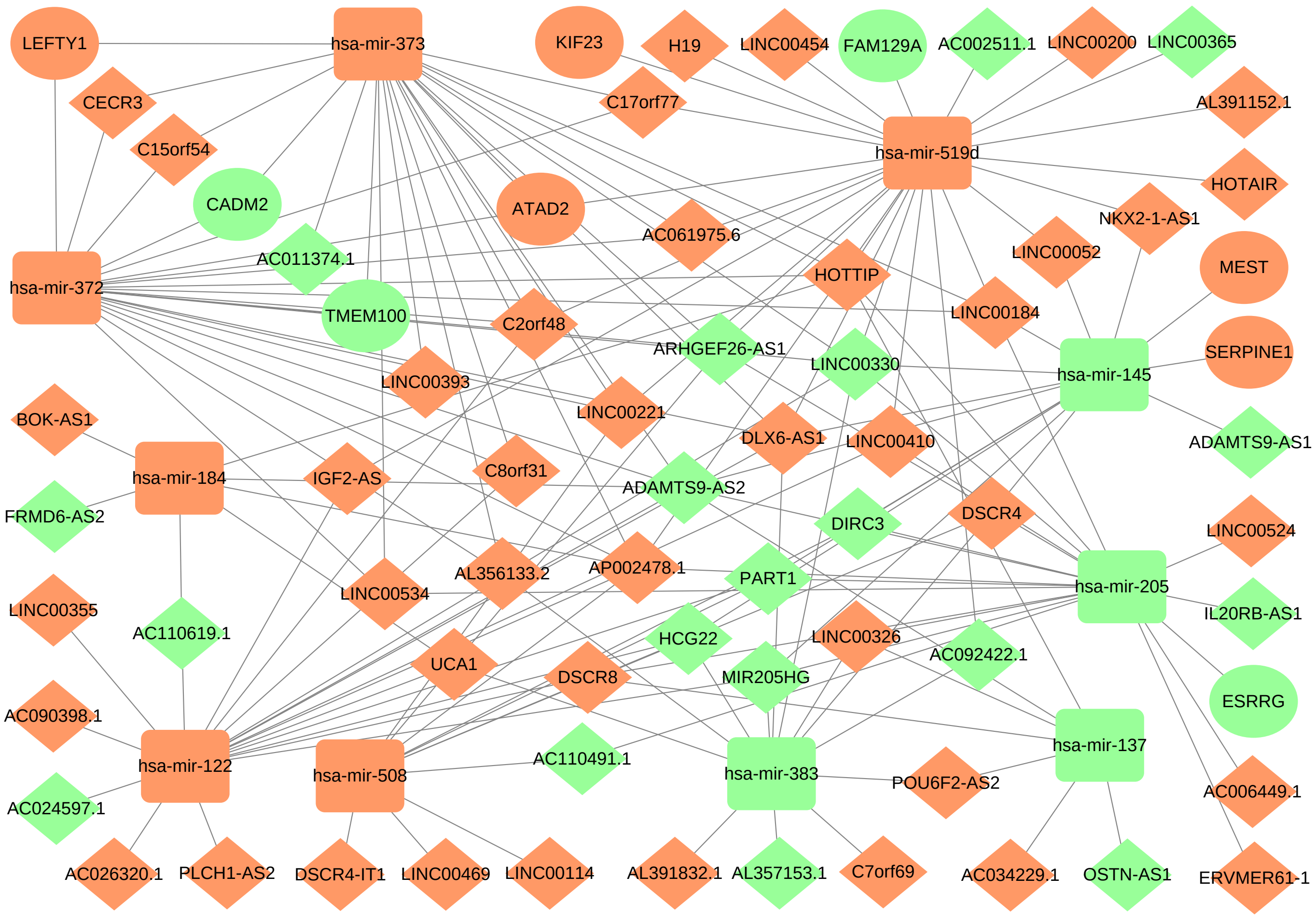
LEFTY1
hsa-mir-373
KIF23
H19
LINC00454
FAM129A
AC002511.1
LINC00200
LINC00365
CECR3
C17orf77
AL391152.1
C15orf54
hsa-mir-519d
CADM2
ATAD2
HOTAIR
NKX2-1-AS1
AC061975.6
hsa-mir-372
AC011374.1
LINC00052
MEST
HOTTIP
TMEM100
LINC00184
C2orf48
ARHGEF26-AS1
LINC00330
hsa-mir-145
SERPINE1
LINC00393
BOK-AS1
LINC00221
DLX6-AS1
LINC00410
ADAMTS9-AS1
hsa-mir-184
IGF2-AS
C8orf31
ADAMTS9-AS2
FRMD6-AS2
DSCR4
DIRC3
LINC00524
AL356133.2
AP002478.1
PART1
hsa-mir-205
LINC00355
LINC00534
IL20RB-AS1
AC110619.1
HCG22
LINC00326
UCA1
AC092422.1
MIR205HG
DSCR8
ESRRG
AC090398.1
hsa-mir-137
hsa-mir-122
AC110491.1
hsa-mir-383
hsa-mir-508
POU6F2-AS2
AC006449.1
AC024597.1
AC026320.1
PLCH1-AS2
DSCR4-IT1
LINC00469
LINC00114
AL391832.1
AL357153.1
C7orf69
AC034229.1
OSTN-AS1
ERVMER61-1

Additional file 4: Figure S3. lncRNA-miRNA-mRNA ceRNA network. Green squares indicate downregulated miRNAs; green circles indicate downregulated mRNAs; green diamonds indicate down-regulated lncRNAs. Orange squares indicate upregulated miRNAs; orange circles indicate upregulated mRNAs; orange diamonds indicate upregulated lncRNAs. The picture is the original version of Fig 1I, which has better readability.
